# Peptides as Versatile Regulators in Cancer Immunotherapy: Recent Advances, Challenges, and Future Prospects

**DOI:** 10.3390/pharmaceutics17010046

**Published:** 2025-01-01

**Authors:** Yu Lei, Jiacheng Liu, Yaowei Bai, Chuansheng Zheng, Dongyuan Wang

**Affiliations:** 1 Department of Radiology, Union Hospital, Tongji Medical College, Huazhong University of Science and Technology, Wuhan 430022, China; ly_00814@163.com (Y.L.); jiacheng6jc@163.com (J.L.); baiyaowei918@163.com (Y.B.); 2 Hubei Provincial Clinical Research Center for Precision Radiology & Interventional Medicine, Wuhan 430022, China; 3 Hubei Province Key Laboratory of Molecular Imaging, Wuhan 430022, China; 4 Department of Pharmacy, Union Hospital, Tongji Medical College, Huazhong University of Science and Technology, Wuhan 430022, China

**Keywords:** peptide-based nanomaterials, peptide vaccines, cancer immunotherapy, drug delivery

## Abstract

The emergence of effective immunotherapies has revolutionized therapies for many types of cancer. However, current immunotherapy has limited efficacy in certain patient populations and displays therapeutic resistance after a period of treatment. To address these challenges, a growing number of immunotherapy drugs have been investigated in clinical and preclinical applications. The diverse functionality of peptides has made them attractive as a therapeutic modality, and the global market for peptide-based therapeutics is witnessing significant growth. Peptides can act as immunotherapeutic agents for the treatment of many malignant cancers. However, a systematic understanding of the interactions between different peptides and the host’s immune system remains unclear. This review describes in detail the roles of peptides in regulating the function of the immune system for cancer immunotherapy. Initially, we systematically elaborate on the relevant mechanisms of cancer immunotherapy. Subsequently, we categorize peptide-based nanomaterials into the following three categories: peptide-based vaccines, anti-cancer peptides, and peptide-based delivery systems. We carefully analyzed the roles of these peptides in overcoming the current barriers in immunotherapy, including multiple strategies to enhance the immunogenicity of peptide vaccines, the synergistic effect of anti-cancer peptides in combination with other immune agents, and peptide assemblies functioning as immune stimulators or vehicles to deliver immune agents. Furthermore, we introduce the current status of peptide-based immunotherapy in clinical applications and discuss the weaknesses and future prospects of peptide-based materials for cancer immunotherapy. Overall, this review aims to enhance comprehension of the potential applications of peptide-based materials in cancer immunotherapy and lay the groundwork for future research and clinical applications.

## 1. Introduction

Cancer is one of the leading public health issues globally, with nearly 20 million new cases and about 9.7 million deaths annually [1,2,3]. Traditional cancer treatment methods, including surgery, radiotherapy, and chemotherapy, have been effective to some extent. Nevertheless, each modality presents distinct limitations. Surgical therapy frequently encounters challenges in completely eliminating tumors, especially when they are located in intricate areas of the body, potentially resulting in recurrence. Radiotherapy’s impact on metastatic and recurrent tumors is constrained and carries the risk of harming healthy tissues. Chemotherapy is highly toxic, damaging not only cancer cells but also healthy ones, which can lead to heightened long-term side effects and an increased risk of secondary malignancies. Therefore, there is an urgent need to develop innovative and more potent strategies for cancer therapy. In recent years, immunotherapy has gained widespread attention as an emerging treatment modality that activates the patient’s immune system to kill tumor cells [4,5,6]. The introduction and broad implementation of cancer immunotherapy have revolutionized clinical practice, having a substantial impact, especially on enhancing patients’ quality of life and extending survival. Cancer immunotherapy is a complex process involving various cell interaction, encompassing both innate and adaptive immunity. Innate immunity includes natural killer (NK) cells, macrophages, dendritic cells (DCs), and neutrophils, which rapidly recognize and respond to tumor cells, inhibiting tumor growth through direct cytotoxicity or cytokine release. Adaptive immunity is more intricate, including post-immunization. Antigen-presenting cells (APCs) capture and internalize peptide antigens, then present antigenic fragments to MHC class I/II molecules, activating CD4^+^ T cells or cytotoxic T lymphocytes (CTLs). Activated T cells release cytokines, triggering CTL responses, while CD4^+^ T cells boost B cell proliferation and IgG production [7]. Cancer immunotherapy can be mainly divided into five categories: vaccines, adoptive cell therapy, immune checkpoint inhibitors (ICIs), monoclonal antibodies, and cytokines [8]. Unfortunately, only approximately 30% of patients respond to these immunotherapeutic approaches due to resistance to cancer immunotherapies, immune evasion, absence of tumor antigens, T cell dysfunction, and an increase in immunosuppressive cells. Consequently, there is an urgent need to improve therapy options and lower recurrence rates [9,10,11].

**Figure 1:**
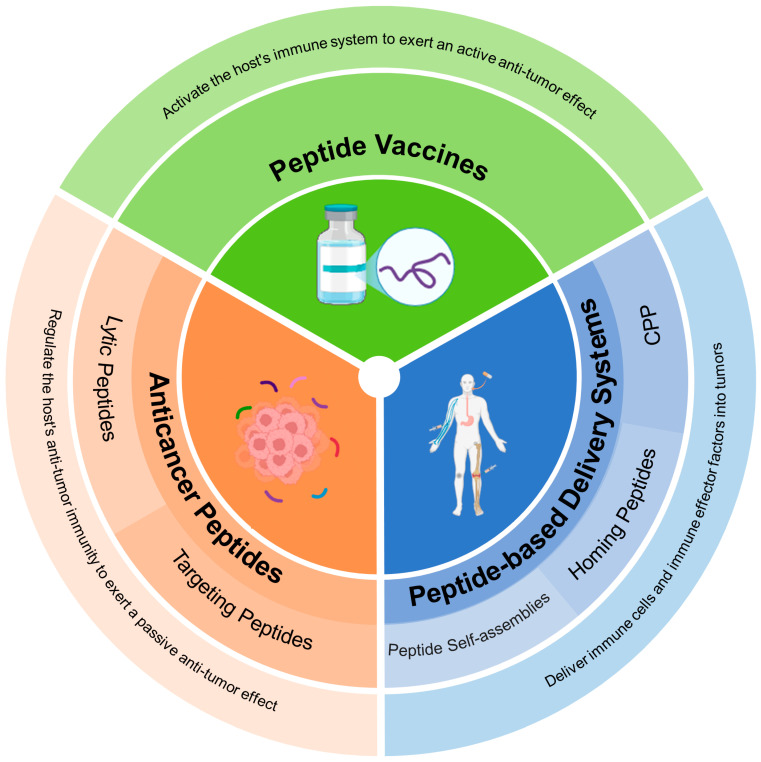
Overview of peptide-based immunomaterials.

Nanomaterials enable targeted drug delivery to tumors or immune organs through their large, modifiable functional groups and high drug-carrying capacity, allowing for efficient biological barrier penetration, precise immunomodulator delivery, and controlled release in response to various stimuli, thus enhancing tumor immunotherapy [12,13]. Peptide-based nanomaterials have emerged as an excellent strategy for cancer therapy, characterized by high specificity, low toxicity, and facile synthesis and modification [14,15]. They fulfill various roles in tumor therapy, including sensing, drug delivery, cell targeting, fate control, deep tumor tissue penetration, and the generation of immune responses for improved anti-tumor therapeutic outcomes [16,17,18,19,20]. For example, they act as vaccine carriers, effectively presenting tumor antigens to activate immune responses for tumor cell recognition and clearance. They also modulate immune cell functions, enhancing the maturation and activation of dendritic cells and stimulating T cell activity, thereby amplifying anti-cancer immune reactions. Furthermore, these peptide-based nanomaterials can also serve as targeted drug delivery systems, directing immunotherapeutic agents to tumor sites to improve efficacy and minimize adverse effects. The diverse mechanisms of action offered by peptide vaccines, anticancer peptides (ACPs), and peptide-based delivery systems introduce innovative strategies for cancer treatment [21,22,23,24]. These peptides bolster the immune system’s ability to recognize and target tumor cells through activation or modulation, thus curbing tumor growth and spread [25,26,27,28,29]. In this review, we systematically introduce the versatility and diversity of peptide-based materials with distinct bioactive properties and discuss their applications in cancer immunotherapy, including peptide vaccines, anti-cancer peptides, and peptide assemblies, as shown in Figure 1. Whether these peptides have the potential to overcome the barriers in current immunotherapy is the largest concern for researches. In this review, we discuss the multiple strategies used to enhance the immunogenicity of peptide vaccines. How anti-cancer peptides show synergistic effects in combination with other immune agents is also reviewed. As for the peptide-based delivery systems for immunotherapy, the roles of cell-penetrating peptides (CPPs), homing peptides and peptide assemblies functioning as vehicles to deliver immune agents or immune stimulators are also discussed. In addition, we also provide the current clinical research on peptide drug conjugates (PDCs) in cancer therapy and analyze the potential for cancer immunotherapy in combination with other immune agents learning from antibody drug conjugates (ADCs). Finally, this article also addresses the challenges and prospective avenues for the evolution of peptide-based materials applied in the clinic. Through an in-depth examination of their interplay with anti-tumor immunity, this review offers novel insights and tactical approaches for combating cancer.

## 2. Peptide Vaccines: Enhancing Immune Response for Cancer Therapy

### 2.1. Concept and Classification of Peptide Vaccines

Tumor peptide vaccines are a type of immunotherapy that aims to bolster both humoral and cellular immune responses against tumors. The composition of peptide vaccines mainly includes peptide antigens and adjuvants. Peptide antigens are either shed from the tumor cell surface or derived from tumor cells and can be recognized by the immune system and trigger an immune response. Adjuvants help to enhance the immunogenicity of the antigen, allowing the vaccine to produce a stronger immune protective effect at lower doses. These vaccines are distinguished by their high specificity, capacity to elicit long-term immune memory, straightforward production process, and superior safety, positioning them as a promising area with extensive market potential [30,31].

The origins of peptide vaccines trace back to the early 1980s with the establishment of methods for creating synthetic peptide vaccines. The foundational approach involved identifying the amino acid sequences of natural antigens, such as those from viruses or their subunits, and pinpointing the peptides responsible for antigenicity. These peptide antigens were then synthesized and evaluated for their capacity to stimulate antibody production, with the goal of selecting peptides that exhibit both immunogenicity and protective qualities for vaccine development. A breakthrough occurred in the early 1990s with the identification of the first human tumor antigen, melanoma-associated antigen 1 (MAA-1), which marked a significant advancement in the incorporation of tumor antigens into cancer vaccines [32]. In 2010, the first vaccine for prostate cancer, Sipuleucel-T [33], was approved for marketing, proving the feasibility and safety of tumor vaccines.

Tumor peptide vaccines target a variety of antigens with different origins. These antigens are primarily classified into two categories: tumor-specific antigens (TSAs) and tumor-associated antigens (TAAs) [16]. TAAs are antigens that are expressed in both tumor and certain normal cells, but their expression levels are typically higher in tumor cells. For instance, HER2 is over-expressed in breast and gastric cancers [34], and MUC1 is expressed in various tumors with altered glycosylation patterns [35]. The advantage of TAAs is that they can serve as common targets for multiple cancer types, facilitating the development of broad-spectrum immunotherapy. However, the expression of TAAs in normal cells may lead to autoimmune reactions during immunotherapy, and tumor cells might evade immune surveillance by downregulating TAA expression, which are the main drawbacks.

In contrast to TAAs, TSAs are unique to tumor cells and are not found in normal cells. They are often produced by mutations in tumor cells, such as cancer-testis antigens (CTAs), which are expressed in various tumors but are negligibly expressed in normal tissues, except for testicular tissue [36]. The superiority of TSAs includes high tumor specificity with low side effects and uniqueness in being easily recognized by the immune system. However, TSAs may exhibit inter-patient heterogeneity, requiring personalized vaccine design, and the diversity of mutations in various cancers can make vaccine design more complex.

Besides directly from tumor cells, peptide antigens can also be derived from the tumor microenvironment. For instance, viral antigens, such as HPV’s E6 and E7 proteins, are vital in virus-induced tumors [37,38]. Additionally, antigens involved in immune regulation, like ARG1, which can modulate the immune response in the tumor microenvironment, represent promising targets for immunotherapy [39]. Tumor peptide vaccines may also aim at proteins on the tumor cell surface, including cell adhesion molecules and growth factor receptors [40,41,42,43], that are pivotal in tumor invasion and metastasis. Furthermore, specific antigens expressed by cells within the tumor microenvironment, such as tumor-associated macrophages (TAMs) and tumor-associated fibroblasts (CAFs), can be leveraged for targeted immunotherapy [44,45].

In summary, the specificity, immunogenicity, and role of target antigens in tumor progression are crucial factors for ideal antigen design with effectiveness and precision of immunotherapy and few side effects. Through the precise targeting of these antigens, tumor peptide vaccines hold promise for delivering more potent therapeutic options to cancer patients.

### 2.2. Mechanisms of Peptide-Based Vaccines

**Figure 2:**
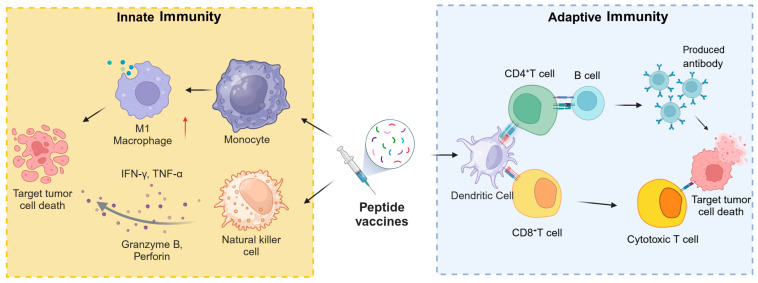
Role of peptide-based vaccines in activating the host’s anti-cancer immunity.

Peptide vaccines engage both innate and adaptive immune responses to form a complex network that identifies and eliminates tumor cells, as depicted in Figure 2.

#### 2.2.1. The Innate Immune System

##### DCs

DCs are pivotal APCs in the immune system, playing an essential role in human immunity. They possess a strong capacity for antigen capture and processing, and the activation of immune cells by secreting various cytokines in the innate immune system. However, in the tumor microenvironment, the function of DCs is often compromised by inhibitory cytokines, leading to their dysfunction and potentially allowing tumor cells to evade immune surveillance. To overcome this challenge, Xi et al. developed a self-healing microcapsule platform that loads various types of antigens (ovalbumin (OVA) protein, mucin 1 (MUC1) peptide, and neoantigen) through a posterior diffusion method and forms microcapsules in a mild encapsulation process. The degradation of microcapsules produces lactic acid, creating an acidic environment that further activates DCs and promotes their maturation and antigen presentation. This method has achieved effective T-cell responses, significant tumor suppression, and anti-metastatic effects in various tumor models, including lymphoma, melanoma, and breast cancer, while also preventing postoperative recurrence [46]. Yan et al. utilized phage display technology to screen a peptide, WH (WPRFHSSVFHTH), that selectively binds to mouse Flt3L-induced Clec9a^+^ DCs or Clec9a highly expressing HEK-293T cells. By conjugating this peptide with the model antigen peptide OVA257-264, they showed great potential in inhibiting tumor metastasis and recurrence in a B16-OVA lung metastatic mouse model [47].

##### NK Cells

NK cells can rapidly identify and eliminate various tumor cells and virus-infected cells. NK cells recognize target cells’ surface MHC I molecules and stress-induced ligands through their inhibitory and activating receptors. Once abnormal cells are detected, NK cells quickly release perforin and granzymes, leading to target cell membrane rupture and cell death. Additionally, NK cells secrete cytokines that regulate immune responses and enhance the activity of other immune cells, playing multiple roles in anti-tumor immunity, including surveillance, defense, and regulation. Therefore, the activation and recruitment of NK cells are essential for tumor treatment [48,49].

Thi-Anh-Thuy Tran et al. developed novel vaccines based on branched peptides and peptide adjuvants, such as the pan-DR epitope (PADRE) and polyinosinic-polycytidylic acid poly(l-lysine) carboxylate (poly-ICLC), which show potential therapeutic effects on glioblastoma [50]. Another screened peptide, SRGPVHHLL, presented by HLA-C*06:02, could strongly activate NK cells expressing KIR2DS1 for immunotherapy [51]. Chisato Yokota et al. developed a vaccine based on Wilms’ tumor gene 1 (WT1) peptides, which activated NK cells by inducing WT1-specific CD8^+^ T cells and CD4^+^ T cells, as well as NK cell infiltration into tumors in the GL261 mouse glioblastoma model [52].

##### TAMs

Macrophages are key cells in the immune system, maintaining tissue homeostasis by engulfing and digesting tumor cells, dead cells, and debris. Macrophages are highly plastic and can polarize into different functional states based on local microenvironmental signals, with the most famous being M1 and M2 macrophages [53]. In anti-tumor mechanisms, macrophages can be activated as M1 type, releasing pro-inflammatory cytokines and reactive nitrogen species, directly killing tumor cells and promoting anti-tumor immune responses, while M2 macrophages are usually associated with tumor promotion and immune suppression. By activating the anti-tumor function of macrophages or inhibiting their tumor-promoting function, the body’s defense against tumors can be enhanced [54]. M2pep, an M2 macrophage binding peptide, YEQDPWGVKWWY) screened by a phage display strategy, has been used to deliver α-peptide (a scavenger receptor B type 1 (SR-B1) targeting peptide) to significantly improve the survival rate of mice in a melanoma mouse model [55]. Another M2pep-based vaccine is also proven to deliver the apoptosis-inducing peptide KLA to TAMs and induce M2 cell apoptosis in a CT-26 colon cancer cell mouse model [56].

##### Neutrophils

Neutrophils are important participants in the innate response of many cancer immunotherapies. When stimulated by chemokines, lipid metabolites, and danger-associated molecular patterns, neutrophils migrate to inflammatory sites and trigger inflammatory responses to combat invading pathogens. Due to the large number of neutrophils in circulation at any given time, they are usually the first cells to arrive at the tumor site. Anti-tumor neutrophils directly eliminate tumor cells by engulfing and releasing cytotoxic substances, while also releasing cytokines and chemokines to regulate immune responses [57,58].

Sara Feola et al. developed a peptide-based oncolytic vaccine, PeptiCab, which uses oncolytic adenovirus (AdCab) carrying programmed death-ligand 1 (PD-L1) checkpoint inhibitors and combines with MHC-I restricted tumor peptides. PeptiCab activates neutrophils by co-activating innate Fcγ and Fcα receptors, giving them an APC-like phenotype, thereby enhancing T cell expansion and activation in melanoma and colon cancer mouse models [59]. Heini M Miettinen et al. identified peptides that can specifically bind to the neutrophil surface molecule CD177 using phage display technology, which could be used for targeted delivery to neutrophils in the tumor microenvironment [60].

#### 2.2.2. The Adaptive Immune System

##### T Cells

Mature T cells play diverse roles in immune responses and are divided into three main types: CTLs, helper T cells (Th cells), and regulatory T cells (Treg cells). Cytotoxic CD8^+^ T cells are responsible for eliminating pathogens and tumor cells. Helper CD4^+^ T cells activate cytotoxic T cells, B cells, and other immune cells, while Treg cells secrete CD4^+^, CD25, and the transcription factor Foxp3 to distinguish endogenous and exogenous substances, reducing the risk of autoimmune reactions. CTLs can identify and kill cells expressing tumor-specific antigens, directly eliminating tumor cells by releasing perforin and granzymes, while promoting the formation of immune memory and producing cytokines such as IFN-γ, which can inhibit tumor cell growth and enhance the anti-tumor activity of other immune cells [61].

Lanqi Cen et al. screened three potential long peptides targeting the MAGE-A4 antigen and combined them with the TLR agonist R848 to develop a multipeptide vaccine. This vaccine activated T cells and enhanced cytotoxic activity in triple-negative breast cancer in mouse models [62]. RNF10, as an E3 ubiquitin ligase is found to play a role in the occurrence and development of tumors. Zeng et al. identified an antigenic peptide (RNF10 uPeptide) through immunoinformatics methods. The epitope can be specifically recognized by CD8^+^ T cells, thereby activating CTLs in the CT26 tumor mouse model [63].

In contrast, Treg cells in the tumor microenvironment mainly secrete immunosuppressive cytokines such as transforming growth factor-β (TGF-β) and interleukin-10 (IL-10) to inhibit the activity of CTL cells and other immune cells. Although Treg cells help maintain immune tolerance and prevent autoimmune reactions, their presence in tumor immunity is usually associated with tumor immune evasion and poor clinical outcomes [64,65]. Therefore, the design of immunotherapy often aims to inhibit the function of Treg cells to enhance the efficiency of anti-tumor immune responses. Teresa Lozano et al. developed a peptide vaccine called P60, which targets the middle region of the Foxp3 protein, inhibiting its homodimer formation and heterodimer interaction with AML1, thus inhibiting the activity of Treg cells [66]. Wang et al. developed a therapeutic peptide vaccine targeting the tumor-associated antigen fibronectin-like protein 2 (FGL2), which is conjugated with the C-terminus of HCMV-IE1mut. In the treatment of glioblastoma (GBM), this vaccine reshaped the tumor microenvironment by reducing the proportion of Treg cells, myeloid-derived suppressor cells (MDSCs), and microglial cells, while increasing the proportion of CD8^+^ T cells and tissue-resident memory T cells (TRMs) [67].

##### B Cells

B cells are white blood cells in the adaptive immune system responsible for producing antibodies. After recognizing specific antigens, they are activated and differentiated into plasma cells, secreting antibodies to neutralize pathogens. In anti-tumor immunity, B cells produce antibodies against tumor-specific antigens, directly inhibiting tumor growth or marking tumor cells to facilitate the recognition and attack by other immune cells [68].

In cancer vaccines, peptides based on B cell epitopes are often used in conjunction with adjuvants to enhance the immune response to immunogenic proteins, prompting B cells to produce polyclonal antibodies. These polyclonal antibodies can recognize a variety of different antigens, contrasting with the specificity of monoclonal antibodies that target particular sites. Additionally, some vaccine strategies employ B cell peptide mimics that can directly bind to specific receptors on tumor cells, preventing receptor dimerization. The anti-cancer mechanism of action of antibodies or their peptide mimics lies in their initial binding to tumor receptors, blocking downstream signaling, and inducing various anti-tumor effects [69].

MUC1 glycoprotein (Mucin 1, also known as MUC1), a large transmembrane glycoprotein is often abnormally increased, and its glycosylation pattern is also altered, leading to the formation of tumor-associated glycoantigens. However, MUC1 is poorly immunogenic. Zhoushan et al. developed a synthetic self-adjuvant multivalent MUC1 glycopeptide vaccine that combines the Tn antigen with a B cell epitope at positions T9, S15, and T16 of the MUC1 tandem repeat peptide to activate B cells and enhance the immune response. In a glioma mouse model, this self-adjuvant trivalent MUC1 vaccine, especially the trivalent vaccine, triggered a strong anti-tumor effect [70]. Guo et al. developed a lot of strategies to enhance the antigen immunogenicity of MUC1 [71,72,73]. For example, they recently developed a multifunctional lipidated protein as a carrier, in which the TLR1/2 agonist Pam3CSK4 was conjugated to the N-terminus of MUC1-loaded carrier protein BSA. This platform elicited robust cellular immunity and significantly inhibited tumor growth [71].

### 2.3. Applications

Tumor peptide vaccines have demonstrated significant promise in therapeutic research for a variety of malignant tumors. Notably, these include gliomas [74,75,76], melanoma [77,78,79], colorectal cancer [80,81,82], lung cancer [83,84], pancreatic cancer [85], ovarian cancer [86], and breast cancer [87,88], among others. Recent research on peptide vaccines for cancer therapy is increasingly focusing on the development of personalized vaccines [22]. The rapid evolution of bioinformatics and computer-assisted technologies is deepening our understanding and leading to the discovery of more target antigens.

In the field of glioma treatment, a first-in-human trial targeting patients with diffuse midline gliomas carrying the histone H3K27M mutation demonstrated that the H3-vac vaccine could induce a specific immune response in patients. In 5 out of 8 patients, an H3K27M-specific immune response was detected, with one patient showing complete remission for over 31 months [74]. Similarly, T. M. Johanns et al. proposed that the neoantigen NeoVax stimulated the expansion of neoantigen-specific effector T cells in patients, extending the survival of patients with glioblastoma [75]. In a phase IIa clinical trial, the SurVaxM vaccine combined with TMZ chemotherapy showed good tolerance and potential clinical benefits in newly diagnosed glioblastoma patients, with 60 patients showing no disease progression after treatment for 6 months, a median PFS of 11.4 months, and a median OS of 25.9 months [76].

GP100, recognized as a melanoma-associated antigen, is a protein that is expressed in melanoma cells or other tumor cells. A pivotal phase III clinical study (NCT00019682) has demonstrated that in patients with advanced melanoma, the concurrent administration of the gp100 vaccine along with interleukin-2 can enhance response rates and extend PFS when compared to the use of interleukin-2 as a monotherapy [77]. Furthermore, a phase III clinical trial conducted in 2004 (NCT00094653) revealed that the combination therapy of Ipilimumab with the gp100 peptide vaccine significantly improved OS in patients with metastatic melanoma who had previously undergone treatment [78].

In addition to GP100, GRP78 has also been found to be highly expressed in various tumor types and is associated with tumor progression. V. A. Brentville et al. demonstrated the existence of a repertoire of responses to citrullinated GRP78 peptides in healthy individuals, with 76% of healthy individuals responding to this peptide, and with citrullinated GRP78 peptides as a potential tumor antigen, targeted vaccines may provide a promising approach for future cancer treatment [79].

In the treatment of colorectal cancer, a phase IIa clinical trial included 103 newly diagnosed colorectal adenocarcinoma patients who, after surgical resection and adjuvant radiotherapy (including TMZ chemotherapy), received injections of the MUC1 peptide vaccine along with TMZ adjuvant chemotherapy. The results proved that the MUC1 peptide vaccine induced an immune response in newly diagnosed colorectal adenocarcinoma patients, with an absolute reduction rate of adenoma recurrence of 38% compared to the placebo [80]. Thymidylate synthase (TS) is a key enzyme that plays an essential role in the DNA synthesis process during tumorigenesis. A phase Ib clinical trial studied the toxicity and bioregulatory activity of a poly-epitope peptide vaccine TS (TSPP) in metastatic colorectal cancer patients, finding that the TSPP peptide vaccine used in conjunction with chemotherapy could induce an immune response and extending survival by 8 months compared to the control group [81]. OCV-C02 vaccine is a peptide vaccine for colorectal cancer, composed of two peptide epitopes that are highly expressed in colorectal cancer. Phase 1 clinical studies showed that the vaccine was safe and well tolerated within a specific dose range, and cytotoxic T lymphocyte (CTL) and delayed-type hypersensitivity (DTH) reactions were observed in the experimental group [82].

In metastatic non-small cell lung cancer (NSCLC), a phase Ib/IIa clinical trial of the universal cancer peptide vaccine UCPVax conducted by Olivier Adotévi et al. found that it achieved longer median OS in patients with immunoresponsive metastatic non-small cell lung cancer [83]. IDO peptide vaccine is an immunomodulatory vaccine targeting immune cells expressing indoleamine 2,3-dioxygenase 1 (IDO1). A phase I clinical trial showed that the IDO peptide vaccine was well tolerated in patients with advanced NSCLC, with two patients showing long-term clinical responses, one of whom had a sustained clinical response 6 years after the first vaccination [84].

WT1 peptide is a product derived from the WT1 (Wilms’ tumor gene 1) gene, which is expressed in various malignancies, including leukemia and solid tumors. Shigeo Koido et al. combined a novel WT1 peptide-pulsed dendritic cell (WT1-DC) vaccine with multiple chemotherapeutic drugs to treat unresectable advanced pancreatic ductal adenocarcinoma, causing significant infiltration of T cells and programmed cell death protein-1^+^ cells in the pancreatic tumor microenvironment (TME), making conversion surgery possible [85]. In addition, Galinpepimut-S (GPS) is also a cancer vaccine targeting the WT1 protein. Galinpepimut-S consists of 4 peptide chains with up to 25 antigenic epitopes, capable of stimulating a strong immune response against the WT1 antigen. A phase I clinical study showed that the combination of galinpepimut-S with nivolumab showed a tolerable toxicity profile and induced immune phenotypic changes and WT1-specific IgG production in patients with WT1-expressing ovarian cancer, with a 1-year PFS of 70% [86].

HER2 in breast cancer is a significant therapeutic target, and the development of the nelipepimut-S peptide vaccine for HER2-positive patients has shown the potential to induce immune responses in clinical trials and may prevent recurrence [87]. Similarly, the E75 vaccine and the GP2 vaccine are two immunotherapeutic peptide vaccines for HER2-positive breast cancer. A meta-analysis pointed out that they can significantly increase the number of CD8^+^ T cells after injection, showing the potential to reduce recurrence and enhance immune responses [88].

These studies not only highlight the potential of peptide vaccines in treating various tumors but also provide new directions for future tumor immunotherapy through personalized vaccine strategies and the exploration of combination therapies. With ongoing advancements in bioinformatics and immunology, research on tumor peptide vaccines promises to bring new hope to cancer patients.

**Figure 3:**
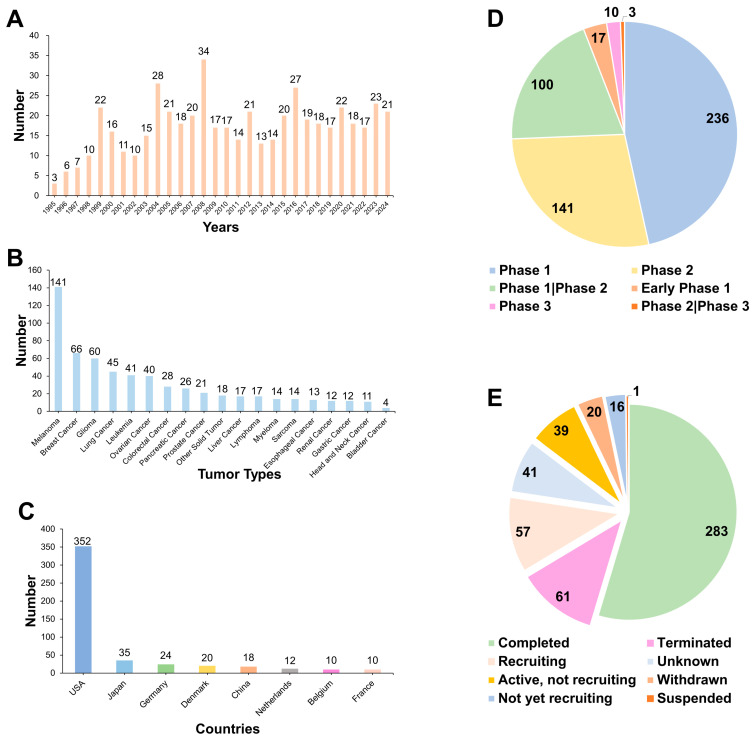
Overview of clinical trials of peptide-based vaccines. (**A**–**E**) Numbers of registered clinical trials grouped by year, tumor type, country, study phase, and status, respectively.

We searched the ClinicalTrials.gov website for peptide vaccine clinical registrations up to October 2024. Figure 3 illustrates the trajectory of peptide vaccine development and the distribution of registered clinical trials, categorized by year, tumor type, country, study phase, and status. Our analysis reveals that a diverse array of tumor types has emerged as focal points in peptide vaccine clinical research, with particular emphasis on those malignancies that exhibit resistance to conventional therapies. Notably, melanoma, breast cancer, glioma, lung cancer, hematological malignancies, ovarian cancer, colorectal cancer, and pancreatic cancer stand out as the most extensively studied categories. These cancer types are typically associated with higher mortality rates and greater resilience to current treatment modalities, underscoring the urgent need for innovative therapeutic approaches.

Regarding the phases of clinical trials, contemporary research on peptide vaccines is primarily focused on Phases I and II. Despite the prevalence of Phase I and II studies, there is a notable scarcity of trials that have advanced to Phase III. Table 1 summarizes all the clinical trials of Phase III peptide-based vaccine registered on the ClinicalTrials.gov website to date. This pattern highlights a significant challenge in peptide vaccine research: the transition from initial human dosage trials to more extensive efficacy validation studies remains a critical hurdle.

### 2.4. Challenges and Prospects

The research and application of tumor peptide vaccines encounter numerous issues and challenges. Firstly, the selection of suitable antigenic epitopes is paramount in the design of these vaccines. It is essential to achieve a delicate balance between immunogenicity and the risk of autoimmune toxicity [99], which involves choosing epitopes that can effectively stimulate immune protective responses without inducing immune attacks on healthy tissues. Secondly, peptide vaccines often exhibit low immunogenicity and are susceptible to enzymatic degradation, resulting in short half-lives and poor stability. This may necessitate frequent injections to maintain efficacy [100,101]. Thirdly, the heterogeneity of tumors constitutes a substantial challenge in cancer treatment. Tumor cells can vary greatly in genotype and phenotype across different regions, over time, and among patients, making it difficult for a single vaccine to target all tumor cells effectively [102,103]. Fourthly, the lack of infiltrating T cells in tumor tissue, or the presence of immunosuppressive factors such as Treg cells and MDSCs, can further diminish the local immune effect of the vaccine and impact overall efficacy [104,105,106,107]. Tumors may also evade immune surveillance through mechanisms like antigen loss and alterations in antigen presentation [108], potentially reducing vaccine efficacy. Fifthly, the choice of adjuvant is crucial for enhancing the immune response. However, there is presently an absence of standardized guidelines for assessing adjuvant effects, and their selection often relies on empirical evidence and trial-and-error approaches [109,110].

Looking ahead, research on tumor peptide vaccines is rapidly advancing towards personalization and precision. A key research direction is the development of personalized vaccines tailored to the specific antigens of a patient’s tumor, such as neoantigens, to enhance the vaccine’s targeting and therapeutic impact [111,112,113,114]. Additionally, combining tumor vaccines with other treatment modalities like radiotherapy, chemotherapy, and immunotherapy (including immune checkpoint inhibitors, oncolytic viruses, and cell therapy) is being explored to augment treatment outcomes [115,116,117,118,119,120]. To improve vaccine stability and bioavailability, researchers are investigating novel vaccine delivery systems, particularly the application of nanotechnology, to enhance the vaccine’s transport capacity and tissue penetration [121,122]. Vaccine design optimization is also a significant research area, encompassing the refinement of vaccine components, such as employing various adjuvants to bolster immune responses [123,124], and improving vaccine formulations to enhance their immunogenicity and therapeutic effects [125]. The development of cyclic peptides represents an emerging research direction aimed at improving the stability and resistance to enzymatic degradation of peptide vaccines [126]. These peptides offer better stability and bioavailability, opening up possibilities for the oral administration of peptide drugs. These research directions not only reflect the advancements in the field of tumor peptide vaccines but also suggest potential breakthroughs on the horizon, with the ultimate goal of improving treatment efficacy and patient quality of life. With ongoing research, tumor peptide vaccines are anticipated to become a vital tool in the arsenal against cancer.

## 3. Anticancer Peptides: Synergistic Effect on Cancer Immunotherapy

### 3.1. Concept and Classification

ACPs are bioactive peptides with antitumor activity, capable of inhibiting tumor cell growth, migration, and invasion through a spectrum of mechanisms [24,127]. The origins of ACPs are diverse, encompassing a wide array of natural biological sources, such as animals, plants, and microorganisms, as well as those synthesized via chemical or biotechnological methods [128,129,130]. The anti-tumor mechanisms of ACPs are multifaceted, ranging from the direct activation of apoptosis pathways to induce tumor cell death [131,132], to the inhibition of tumor neovascularization [133,134], disruption of tumor cell membrane integrity [135,136], and enhancement of immune cell activity to bolster the body’s anti-tumor immune response [137,138]. Based on their immune-activating mechanisms, ACPs can be categorized into two main groups: targeted peptides that inhibit immune-related signal pathways aberrant in cancer cells such as PD-1/PD-L1 interaction, cGAS-STING pathway, and CD47/SIRPα; and lytic peptides, which form pores in cancer cell membranes to induce cell necrosis or apoptosis, and the cell fragments can act as tumor antigens to trigger the immune response.

### 3.2. Mechanisms of ACP-Induced Immune Response

Targeted peptides usually interrupt specific pathways associated with anti-tumor immunity, leading to tumor cell death. Numerous studies have focused on the PD-1/PD-L1 immune-inhibitory pathway [139,140,141,142,143,144,145]. Zhang and colleagues employed a “three-dimensional molecular evolution” screening strategy to identify an efficient and versatile peptide, termed TAP. This peptide possesses self-assembly capabilities and can effectively block the PD-1/PD-L1 axis, thereby activating T cells and NK cells. It also inhibits the formation of the Rbm38-eIF4E complex and activates p53. In vivo experiments have demonstrated significant inhibitory effects on both subcutaneous tumors in mice and patient-derived xenograft tumors [145]. Yang and colleagues investigated a fish oil-based microemulsion for the oral delivery of the PD-1/PD-L1 blocking peptide, OPBP-1. The in vivo studies revealed that this microemulsion enhances the infiltration of CD8^+^ T cells into tumors, increases the secretion of IFN-γ, and markedly suppresses the growth of mouse colon cancer cells (CT26) [144].

**Figure 4:**
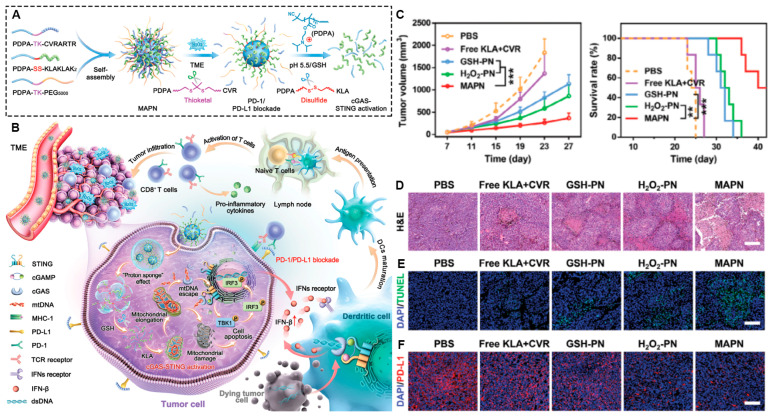
MAPN—mediated activation of the cGAS-STING Ppthway and PD-1/PD-L1 blockade for cancer immunotherapy. (**A**,**B**) Schematic illustration of multi-stimulus activatable peptide nanodrug (MAPN) activating the cGAS-STING pathway by promoting mtDNA leakage and blocking the PD-1/PD-L1 pathway to initiate robust and durable anti-tumor immune responses for cancer immunotherapy. (**C**–**F**) MAPN treatment could significantly inhibit tumor growth, promote tumor necrosis, and prolong the survival of B16F10 tumor-bearing mice. Adapted with permission from [146], copyright 2024 Advanced Science, published by Wiley-VCH GmbH. (** *p* < 0.01, *** *p* < 0.001).

Besides the PD-1/PD-L1 pathway, the cGAS-STING pathway is another critical signal transduction route in cancer immunotherapy. It consists of cGAS (cyclic GMP-AMP synthase) in the cytoplasm and the stimulator of interferon genes (STING) in the endoplasmic reticulum, playing a pivotal role in both innate and adaptive immunity. Tumor-derived antigens in the tumor microenvironment can activate the cGAS-STING pathway, prompting the production of type I IFN, which bolsters dendritic cell maturation and mediates T cell activation, thus igniting anti-tumor immunity. A recent study introduced a multi-stimulus-responsive peptide nano-drug (MAPN), which achieves site-specific release and simultaneously activates the cGAS-STING pathway while blocking the PD-1/PD-L1 pathway. This dual action triggers a potent and sustained T cell anti-tumor immune response, effectively curbing the growth, recurrence, and metastasis of malignant tumors [146], as shown in Figure 4.

CD47/SIRPα is a vital molecule in the immune system, playing an essential role in the regulation of macrophage phagocytic activity. Tumor cells often overexpress CD47 on their surfaces, thereby suppressing macrophage function through the CD47-SIRPα pathway, evading phagocytosis, and thus achieving immune escape. Gao et al. utilized a high-throughput phage display library to screen for a novel peptide, Pep-20, which targets CD47 and disrupts the CD47/SIRPα interaction. This enhances macrophage-mediated phagocytosis and induces effective anti-tumor immune responses, thereby inhibiting tumor growth in immunocompetent tumor-bearing mice. The study also discovered that the D-amino acid derivative of Pep-20, Pep-20-D12, exhibits a strong synergistic anti-tumor effect when combined with radiotherapy [147].

TIGIT is an emerging immune checkpoint molecule expressed on NK and T cells, competing with the co-stimulatory receptor CD226 for the shared ligand PVR to transmit immune-inhibitory signals [148]. Recent research has indicated that TIGIT is overexpressed in many tumors compared to PD-1, particularly in tumors resistant to anti-PD-1 therapy, highlighting TIGIT’s potential as a therapeutic target. Gao and colleagues synthesized a TIGIT D-peptide using a hydrazine-based in situ chemical ligation method and selected a D-peptide, DTBP-3, that can occupy the binding interface of TIGIT and its ligand PVR through mirror phage display technology. DTBP-3 can penetrate tumor tissue and significantly inhibit tumor growth and metastasis in anti-PD-1-resistant tumor models in a CD8^+^ T cell-mediated manner [149].

**Figure 5:**
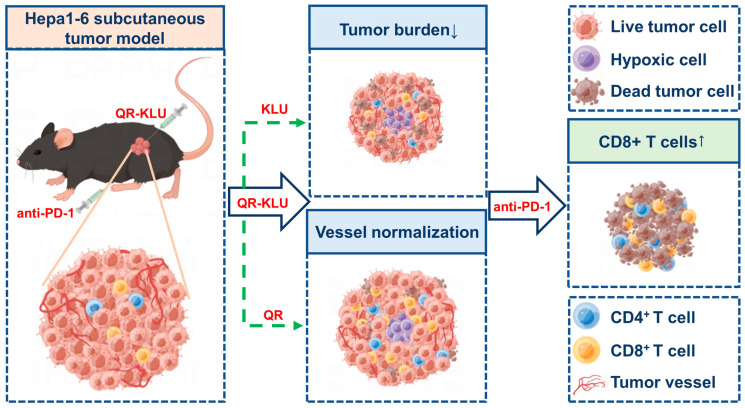
A VEGFR-targeting peptide VEGF125-136 (QR) was conjugated with a lytic peptide (KLU) to form a peptide–drug conjugate QR-KLU, QR-KLU and anti-PD-1 antibody demonstrating a strong synergistic effect in promoting the activation of intratumoral CD8^+^T cells, reducing the expression of immune-inhibitory factors, and increasing the expression of immune-stimulatory factors. The green arrows denote the peptide QR-KLU, which is comprised of the sequences QR and KLU. The KLU component induces cytotoxicity in tumor cells through membrane disruption, thereby decreasing tumor burden, while the QR component exhibits anti-angiogenic properties by targeting the vascular endothelial growth factor receptor (VEGFR), facilitating the normalization of tumor vessels. The blue-bordered arrows with white fill illustrate that, under the influence of QR-KLU in conjunction with the anti-PD-1 antibody, the tumor undergoes necrosis, vascular normalization is achieved, and the immune system is activated. Adapted with permission from [152], copyright 2024 Springer Nature.

Lytic peptides have direct lytic effects on the tumor cell membrane and kill cancer cells through necrosis, producing tumor-associated antigens, which synergistically enhance anti-cancer effects with other immune therapies. For example, Kim et al. found that LHRH-R-targeted lytic peptide could enhance cancer immunotherapy in combination with PD-1 antibodies by increasing the population of CD8^+^ T cells, NK cells, dendritic cells, and macrophages in tumors. Melittin is a widely used lytic peptide for cancer therapy. Tang and colleagues engineered a tumor microenvironment (TME)-responsive MnO2-melittin nanoparticle (M-M NPs) that activates the cGAS-STING pathway, promotes the maturation of antigen-presenting cells, and initiates systemic anti-tumor immune responses [150]. Our research team has developed a peptide-drug conjugate (PDC) by linking a VEGFR-targeting peptide, VEGF125−136 (sequence QKRKRKKSRYKS), with a lytic peptide (KLUKLUKKLUKLUK, named KLU). In a VX2 tumor-bearing rabbit model, this PDC exhibited superior in vivo antitumor efficacy over the conventional drug DOX and effectively suppressed tumor-associated angiogenesis while also showing favorable safety profiles [151]. Moreover, we demonstrated that the combination of this PDC with an anti-PD-1 antibody had a potent synergistic effect, enhancing the activation of CD8^+^ T cells within the tumor microenvironment, diminishing the expression of immunosuppressive factors, and upregulating the expression of immunostimulatory factors, as shown in Figure 5 [152]. Additionally, in another study, we explored the therapeutic potential of combining the lytic peptide LTX-315 with radiofrequency ablation and doxorubicin chemotherapy to prevent tumor recurrence following thermal ablation in medium to large hepatocellular carcinomas (HCC) [153].

### 3.3. Challenges and Prospects

ACPs hold great promise as therapeutic agents for cancer treatment and immunotherapy. However, they have also met several challenges for clinical translation. Firstly, the large-scale production and purification of bioactive peptides present significant technical hurdles, coupled with high costs and the challenge of maintaining product purity [24]. Secondly, these peptides, like other peptide-based drugs, grapple with issues of stability and bioavailability [154,155,156]. Thirdly, delivering these peptides effectively to the target site, enhancing cellular uptake, and mitigating immune responses pose considerable technical obstacles [157,158]. Lastly, while some studies have delved into the potential anti-tumor mechanisms of these peptides, including their immunoregulatory effects, the precise principles and pathways require further elucidation.

To address these challenges, future research will concentrate on the discovery of novel bioactive peptides. This includes leveraging high-throughput screening techniques, bioinformatics analysis, and proteomics to pinpoint potential bioactive peptide sequences. In-depth structural and functional studies will be pivotal in deciphering the bioactivity mechanisms of these peptides, which is essential for devising new therapeutic strategies. Moreover, advancements in production techniques, such as genetic engineering, enzyme engineering, and cell culture technology, will enhance yield and purity while driving down costs. The deployment of effective delivery systems, like nanotechnology and liposomes, will ensure the precise delivery and in vivo stability of peptides [159]. Exploring drug combination strategies, including the use of peptides in conjunction with other therapeutic agents, will augment treatment efficacy and diminish side effects [160,161]. Collaborative interdisciplinary research will spur innovation and progress in the field of bioactive peptides. Ultimately, as research progresses, the regulation and safety assessment of these peptide drugs will emerge as focal points to ensure their safety and regulatory compliance in clinical settings.

## 4. Peptide-Based Delivery Systems for Cancer Immunotherapy

**Figure 6:**
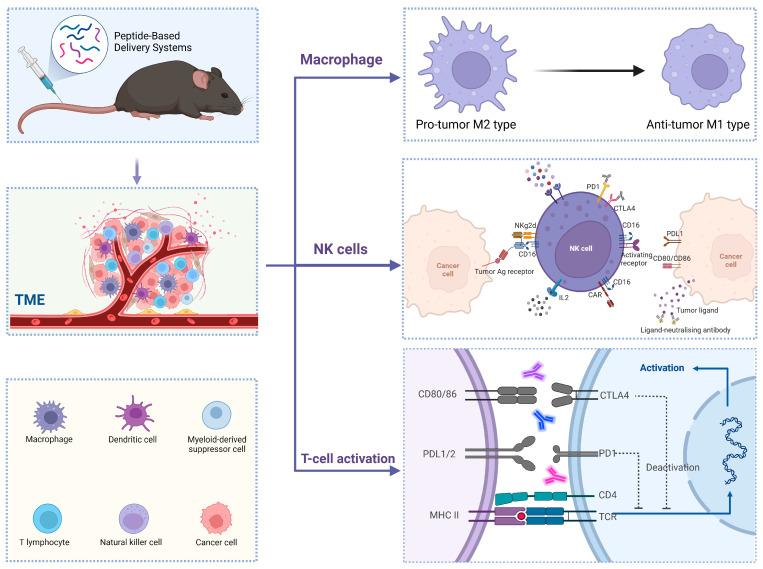
Tumor immune response induced by peptide-based delivery systems. A targeted peptide–antigen system incorporating immune adjuvants was administered intravenously to murine cancer models. Upon entering the bloodstream, the system traverses to the tumor microenvironment (TME) and undergoes decomposition. Antigenic peptides are subsequently internalized by antigen-presenting cells (APCs) and presented to naïve T cells. This interaction leads to the activation of T cells, which then recognize tumor antigens and secrete cytokines. Concurrently, the released immune adjuvant is phagocytosed by macrophages, inducing a phenotypic shift towards antitumor M1 tumor-associated macrophages (M1-like TAMs). Simultaneously, activated natural killer (NK) cells facilitate the recruitment of monocytes and collaborate with T cells and macrophages to target and eliminate tumor cells.

In addition to their direct impact on the immune system, peptides can also act as vehicles to deliver immunologically active drugs to the tumor site via cell penetrating peptides, tumor homing peptides, or self-assembling peptide materials. Figure 6 summarizes the tumor immune response induced by peptide-based delivery systems.

### 4.1. Cell-Penetrating Peptides (CPPs)

CPPs are short peptide chains with unique structures and functions. They are classified into three types based on their origins: naturally occurring, synthetic, and chimeric. Table 2 lists some common CPPs.

The mechanisms by which they deliver cargo primarily include direct penetration, endocytosis, and the formation of transient membrane pores. Direct penetration is an energy-independent process where CPPs traverse the cell membrane to deliver drugs or other molecules into the cell. Endocytosis involves the cell membrane folding inward to form vesicles that carry CPPs and their cargo into the cell. This can occur through several pathways, including clathrin-mediated endocytosis, caveolae-mediated endocytosis, and macropinocytosis. Additionally, CPPs may create temporary pores in the cell membrane, facilitating the transmembrane transport of drugs. Some CPPs facilitate drug transport by increasing the fluidity of the cell membrane, while others may induce the formation of transient membrane protrusions or lamellipodia to assist in cargo delivery [172]. It is important to note that the specific mechanism of CPP penetration may depend on various factors, such as the nature of the cargo, cell type, membrane composition, and peptide concentration [173]. Although the positive charge of CPPs aids in their interaction with the negatively charged cell membrane, promoting the transmembrane transport of therapeutic molecules, the precise mechanism of CPP penetration remains an active area of research and is not yet fully understood.

CPPs conjugated with various cytotoxic molecules and immunotherapeutic drugs have been extensively utilized in cancer treatment, often demonstrating sufficient targeting effects in tumor cells to precisely and efficiently eliminate them. By delivering molecules that activate various immune cells, effective anti-tumor immunity can be enhanced [174].

Yuri Fujioka et al. proposed VP-R8, a cell-penetrating D-octaarginine-linked co-polymer of N-vinylacetamide and acrylic acid, which has been shown to significantly enhance the efficiency of dendritic cell-based vaccines in priming robust anti-tumor immunity. Notably, intranodal injection of this compound has been found to markedly reduce tumor growth, highlighting its potential as a valuable additive in dendritic cell-based immunotherapy [175]. Sangho Lim et al. identified and characterized a novel high-quality CPP named dNP2. This peptide has the ability to more effectively and rapidly deliver conjugated EGFP protein to APCs, such as macrophages and dendritic cells, compared to other immune cells [176], thereby improving the prospects of DC vaccines.

In order to enhance the anti-tumor activity of T cells, Madiha Derouazi et al. used a CPP (Z12) to achieve cytoplasmic delivery of tumor antigens, which can then be processed and presented by MHC class I molecules, promoting persistent CD8^+^ T cells with strong cytotoxic function, extending survival in three robust tumor models [177]. Similarly, Wang et al. employed the MTS (AAVLLPVLLAAP) fusion protein to deliver TRP2 peptides into DCs, thereby prolonging antigen presentation. This strategy induced TRP-2 specific CD8^+^ T cells and significantly enhanced the anti-tumor response in the B16 tumor model, as well as preventing lung metastasis [178].

The blood–brain barrier (BBB) and the complex tumor immunosuppressive microenvironment present significant challenges in combating brain tumors such as glioblastoma. To address this issue, Zheng et al. modified NP-siRNA with the cell-penetrating peptide tLyp-1 and fused it with a checkpoint inhibitor monoclonal antibody (aNKG2A), forming a dual-functional and siLSINCT5-loaded dendritic nano-particle (tLyp/aNKNP-siRNA) that promotes anti-tumor immunity by releasing T cells and NK cells. The effectiveness of this approach has been thoroughly confirmed in both in vitro cell cultures and in vivo animal models [179].

Additionally, gene therapy, which targets tumor treatment in two-thirds of cases, represents an effective approach to tumor management. A recent study utilized the CPP and dNP2 for siRNA delivery to knock down chitinase-3-like-1 (Chi3l1), a negative regulator of Th1 differentiation and CTL function [180]. Administered nasally to mice, the dNP2-siChi3l1 complex significantly inhibited the metastasis and growth of B16F10 melanoma cells in the lungs, with a notable increase in Th1 cell and CTL levels. This outcome supports the potential of CPP coupling strategies. Messenger RNA (mRNA)-based gene therapy in cancer holds great promise, and modification with CPPs can enhance delivery efficiency, offering a beneficial strategy to amplify the therapeutic impact of mRNA gene therapy [181,182].

Despite their potent targeting capabilities, CPPs may also accumulate in off-target cells or tissues, including the liver or kidneys, potentially causing toxicity [183]. Therefore, the pharmacokinetics, biodistribution, and hepatic and renal toxicity of CPP conjugates must be meticulously evaluated to prevent adverse effects.

### 4.2. Homing Peptides

Tumor-homing peptides are specialized molecules that target tumors or the tumor microenvironment, including tumor neovasculature and the extracellular matrix, with specificity for tumor-associated surface markers such as membrane receptors [184]. Their tumor-targeting capabilities and high permeability make them ideal for guiding therapeutic agents into tumor tissue or the vascular system, thereby playing a crucial anti-tumor role while minimizing side effects and maximizing drug concentration at the tumor site [185].

In the realm of tumor immunotherapy, homing peptide targeting strategies have been employed to modify cytokines or immune cells to activate the immune response. Kai Temming and colleagues synthesized a fusion protein (RGD-IL-12) that connects mouse IL-12 to RGD4C (integrin peptide ligand). This fusion protein retains the immune-stimulating activity of IL-12, effectively activates NK cells, and induces NK and T cells to produce IFN-γ. In corneal angiogenesis assays, RGD-IL-12 demonstrated more effective inhibition of blood vessel growth than native mIL-12, nearly completely inhibiting bFGF-induced angiogenesis. In a neuroblastoma model (NXS2 model), RGD-IL-12 also exhibited enhanced anti-tumor effects [186]. Additionally, Schraa and colleagues modified anti-CD3 antibodies with RGD to redirect cytotoxic T cells [187]. The resulting RGD anti-CD3 conjugate showed a high affinity for αvβ3 and maintained its ability to bind to cytotoxic T lymphocytes. In vitro experiments indicated that this conjugate could induce CTL-mediated lysis of HUVEC cells in an RGD-dependent manner, suggesting its potential to induce immune-mediated vascular damage and tumor cell killing.

Furthermore, homing peptides can target tumors and activate local immune cells. Gao and colleagues utilized selenium-containing nanoparticles (NPs) containing tumor-targeting peptide-modified polyethylene glycol (PEG-RGD) for systemic administration to deliver the chemotherapeutic drug doxorubicin (DOX) to the tumor site. Combined with radiation stimulation, the selenium-containing NPs were oxidized into selenic acid, effectively enhancing NK cell function and exerting synergistic anti-tumor effects and immune regulatory activity [188].

PDCs, consisting of homing peptides, linkers, and toxic drugs, have attracted great attention in the drug industry due to their similarity to ADCs. Currently, the most promising PDCs used for clinics are radiolabeled PDCs approved for disease diagnosis or therapy [189]. Other PDCs conjugated with toxic drugs are under clinical study. For example, AEZS-108 (zoptarelin doxorubicin), a cytotoxic hybrid molecule comprising doxorubicin covalently linked to a LHRH analog, has been evaluated in a Phase II trial in men with metastatic castrate-resistant prostate cancer who had progressed after taxane-based chemotherapy [190]. ANG1005 (Angiopep-2), consisting of three paclitaxel molecules covalently linked to Angiopep-2, is designed to facilitate crossing the blood–brain and blood–cerebrospinal barriers and penetrating malignant cells via the LRP1 transport system, which had been evaluated in an open-label phase II study (NCT02048059) in adults with recurrent breast cancer brain metastases [191]. TH1902 is a docetaxel–peptide conjugate designed to selectively target cancer cells overexpressing sortilin (SORT1) receptor. In early 2021, the FDA granted TH1902 fast-track designation for monotherapy in treating sortilin-positive recurrent advanced solid tumors that are resistant to standard therapies [192]. Recent studies revealed that TH1902 could trigger the cGAS/STING pathway and potentiate anti-PD-L1 immune-mediated tumor cell killing in murine B16-F10 melanoma syngeneic tumor model [193]. Bicyclic toxin peptides (BTPs), cyclization with three cysteine residues that form disulfide bonds, showed potential for the development of PDCs due to their high target affinity, serum stability and good safety. In 2021, Bicycle Therapeutics announced three investigational BTPs (BTCs) for Phase II/III clinical trials, including BT1718 [194], targeting membrane type I matrix metalloproteinase (MT1-MMP) with toxic drug DM1, BT5528 [195] targeting EphA2, and BT8009 [196] targeting nectin-4. With the application of ADCs in combination with immune agents to enhance cancer immunotherapy for different cancer patients, there will be new discoveries of PDCs in sensitizing immunotherapy [197,198,199].

### 4.3. Supramolecular Peptide Assemblies as Immunomaterials for Cancer Therapy

**Figure 7:**
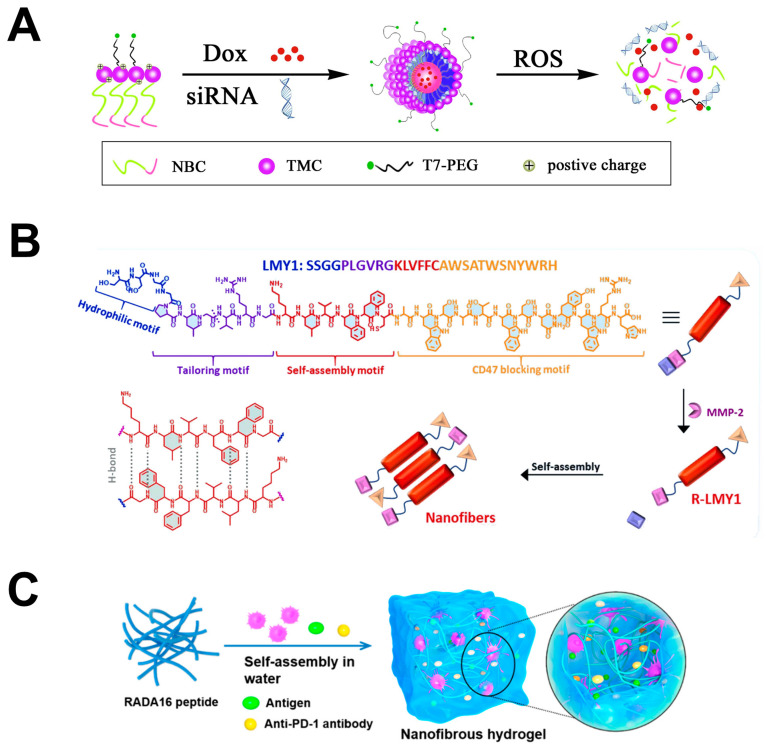
Schematic illustration of representative forms of peptide self-assemblies. (**A**) Nanoparticles. Dox, doxorubicin; ROS, reactive oxygen species. Reprinted with permission from [205], copyright 2019 Elsevier B.V. (**B**) Nanofibers. Reprinted with permission from [206], copyright The Royal Society of Chemistry. (**C**) Hydrogels. Reprinted with permission from [207], copyright 2018 Spring Nature.

Peptide self-assemblies represent an advanced class of nanostructures that spontaneously form through non-covalent interactions such as hydrogen bonds, hydrophobic interactions, and electrostatic forces. This self-assembly process is a sophisticated method for creating highly ordered structures in a controlled, spontaneous manner, modulated by factors like temperature, pH, and concentration [200]. These assemblies are distinguished by their stability, diversity, biocompatibility, functionality, stimulus responsiveness, and tunability, positioning them as highly promising candidates in the biomedical field, particularly for drug delivery systems [201]. Common forms of peptide self-assemblies include nanoparticles, nanofibers, nanotubes, nanospheres, and hydrogels [202,203,204], as shown in Figure 7. These peptide self-assemblies serve as drug carriers to safeguard drug molecules, enhance their stability and bioavailability, and facilitate targeted delivery. Table 3 illustrates various types of self-assembled peptides utilized in the research and application of anti-tumor immunotherapy.

In the realm of anti-tumor immunotherapy, peptide self-assemblies are pivotal. They are designed to specifically target and block immune-suppressive pathways while simultaneously activating immune cells, including NK cells [212,223], DCs [220,222], TAMS [214,215], and CTLs [208,209,218]. For instance, David et al. developed a novel injectable peptide hydrogel termed STINGel for the controlled release of STING agonists, which activates the STING pathway, bolsters innate immunity, and significantly improves the survival rate in mice with head and neck cancer [222]. In addition, significant progress has been made in the development of peptide self-assemblies targeting the PD-1/PD-L1 pathway, which plays a pivotal role in tumor immune evasion [205,211,212,217,218,220]. Zhang et al. devised a peptide nanofiber system that actively delivers drugs via a co-assembly method, integrating immune checkpoint blockades with sonodynamic therapy to effectively inhibit tumor growth, impacting both primary and distant tumors [217]. Compared to nanofibers, nanoparticles represent a class of peptide self-assemblies that have garnered extensive research attention and practical application. Wan et al. have explored an ROS-responsive nanoparticle system, enhanced with T7 modifications for targeted binding to transferrin receptors overexpressed on breast cancer cells. This strategy facilitates enhanced cellular internalization and the co-delivery of siRNA-PD-L1 along with doxorubicin (Dox). The elevated ROS levels within the cancer cells act as a trigger for the swift release of Dox, inducing apoptosis specifically in breast cancer 4T1 cells [205]. In a parallel approach to breast cancer therapy, Wang et al. have introduced a personalized nanogel-based cancer vaccine, PVAX, which, upon near-infrared laser activation, effectively stimulates dendritic cell maturation and elicits an influx of tumor-infiltrating cytotoxic T lymphocytes. The synergistic application of JQ1, a BRD4 inhibitor, mediates the PD-L1 checkpoint blockade, thereby initiating a potent anti-tumor immune response that curbs the recurrence and metastasis of breast cancer tumors [220]. Indoleamine 2,3-dioxygenase 1 (IDO-1), an enzyme overexpressed in tumor tissues, converts tryptophan to kynurenine, depleting tryptophan in the tumor microenvironment and suppressing T cell function, thus facilitating tumor immune evasion [223]. Li et al. introduced a novel peptide-based self-assembled nanoparticle that co-assembles a pentapeptide containing 4-aminoproline and its derivative, including the IDO-1 inhibitor NLG919. Combined with external γ-ray radiation therapy, it can trigger a cascade of immunogenic cell death (ICD) in tumor cells and deplete tryptophan by inhibiting IDO-1, reversing the immunosuppressive tumor microenvironment [213].

### 4.4. Challenges and Prospects

Delivery systems should focus on achieving precise targeting and ensuring stability. CPPs demonstrate a remarkable ability to infiltrate various tissues, yet the underlying mechanisms of their cellular internalization remain obscure. This complexity poses challenges in the design and optimization of these peptides. Furthermore, the cell membrane penetration by CPPs lacks the desired specificity for particular cells and tissues, which can limit their effectiveness in targeted delivery. Additionally, concerns about the cytotoxic effects of CPPs at high concentrations underscore potential safety issues in clinical applications [224].

Homing peptides, while capable of specifically homing on tumor cells or vasculature, might still fall short in selectivity, resulting in non-specific cellular uptake or off-target effects [225]. Furthermore, there is a risk that certain targeting peptides could share sequence homology with receptors present in healthy tissues, which could lead to undesirable side effects [226].

Peptide hydrogels have garnered significant interest as a prominent subject within the realm of peptide self-assembly research. Peptide hydrogels can directly stimulate the activation of immune cells [227] and also serve as carriers for various immunotherapeutic agents, including vaccines, immune cells, cytokines, and immune checkpoint inhibitors, to enhance the efficacy of cancer immunotherapy [220]. However, the stability of these structures, formed by non-covalent interactions, is relatively weak, posing challenges for the formation of long-lasting stable hydrogels in clinical settings [8]. Moreover, achieving length control and consistent morphology is difficult, which may lead to heterogeneity in immunotherapeutic effects. Additionally, the precise immune mechanisms of many supramolecular peptide platforms remain undiscovered.

Future research directions may involve exploring the relationship between the structural, physical, and mechanical properties of peptide-based delivery systems and immune-related mechanisms, integrating with other treatment modalities such as CAR-T cell therapy, developing shear-thinning injectable hydrogels for local tumor administration, and leveraging machine learning for biomaterials development. These research avenues are anticipated to further advance the application of peptide-based delivery systems in cancer immunotherapy delivery.

## 5. Conclusions

Despite the successful application of cancer immunotherapy among a wide range of cancer types, only a minority of patients benefit from these therapies due the complexity of the immune system. The barriers to effective immunotherapy included lack of tumor antigen processing and presentation, ineffectiveness of infiltrating immune cells, inhibited trafficking and infiltration of immune cells to the tumor site, et al. [228]. To address these problems, therapeutic advances have rapidly emerged and received great interest. Peptide-based cancer immunotherapy has attracted considerable attention due to its excellent biocompatibility, high target affinity, ease of modification, and facile synthesis. Meanwhile, the diverse function of peptides in cancer therapy exhibited great potential in regulating cancer immune environment. To improve the tumor antigen presentation, peptides that selectively binds to DCs could be conjugated with peptide antigens to activate tumor immune response. The efficacy of peptide vaccines can be improved by the use of novel adjuvants, neoantigens, nano-delivery systems, and combination therapies [30]. The selection of appropriate antigens with enhanced immunogenicity is the key to addressing tumor heterogeneity and overcoming the immunosuppressive tumor microenvironment. Nowadays, many TSAs and TAAs have been developed to enhance tumor immune response for different cancer patients. Among these antigens, personalized peptide vaccines are chosen to complement pre-existing host immunity by the use of multiple cancer peptides, which have been proven to induce strong and rapid cancer immunotherapy in many cancer patients [229]. Other peptide vaccines, such as those targeting NK cells, have been investigated to enhance T cell expansion and activation of T cells in animal models. Despite the numerous strategies for CTL activation, the exhausted CTL in the tumor immune microenvironment becomes the key process for the resistance of cancer immunotherapy. Peptide vaccines targeting B cells with adjuvants, such as MUC1-targeting peptide vaccines, have been proven to enhance the immune response in both animal models and cancer patients by producing polyclonal antibodies.

Another strategy to overcome the resistance of cancer immunotherapy is the combination of anti-cancer targeting peptides with other immune drugs. For example, the combination of lytic peptides and PD-1 antibody has been validated to enhance the therapeutic effects on tumor animal models. This strategy might provide a new approach for enhancing cancer immunotherapy. Due to the high target affinity, peptide inhibitors have been developed to disrupt the PD-1/PD-L1 axis, the cGAS-STING pathway, CD47/SIRPα pathway et al., which become a promising complement for cancer immunotherapy, and some of what could penetrate tumor tissue and significantly inhibit tumor growth and metastasis in anti-PD-1 resistant tumor models. 

Peptides also face the problem of low target binding affinity with limited anti-cancer effects. Most importantly, the stability and bioavailability of peptides are pivotal factors that currently limit their clinical utility. Peptide self-assemblies typically form through non-covalent interactions which are naturally weak and may not be stable enough in blood circulation. Meanwhile, many supramolecular peptides tend to be heterogenous and lack controllability, which could result in various immunotherapeutic outcomes.

In recent years, high-throughput screening technologies have developed rapidly and have been widely used for screening for therapeutic peptides with high binding affinities and improved physicochemical properties, including phase display [230], one-bead-one-compound (OBOC) method [231], mRNA display [232], split-intein circular ligation of peptides and proteins (SICLOPPS), et al. [233]. With the aid of these techniques, versatile peptide libraries including peptide antigens, anti-cancer peptides, or self-assembling peptides will be developed which will accelerate the clinical application of peptide-based cancer immunotherapy. As for the poor stability of peptides, many chemical methods have been widely developed such as peptide cyclization, incorporating unnatural amino acids, or covalent modification at N-terminus or C-terminus of peptides et al. These modifications have been proved to improve serum stability and prolong their in vivo half-life. Meanwhile, the current developments in machine learning would also be a potential expansion area in the peptide-based cancer immunotherapy where the potential peptide sequence hits with strong immunomodulatory effects can be predicted by silico models. In summary, peptide-based biomaterials can generate and modulate interactions with immune cells or cancer cells. More in-depth research on the immune-modulation mechanisms of peptides will aid in the design of novel immune-instructive peptide-based nanomaterials and translate their use for clinical cancer therapies.

## Figures and Tables

**Table 1:** Clinical trials of peptide vaccines of phase III and above.

Clinical Trial ID	Start Date	Status	Antigen	Type of Cancer	Enrolled Participants	Reference
NCT00019682	1999	Completed	gp100	Melanoma	185	[89]
NCT01989572	2000	Completed	Tyrosinase Peptide	Melanoma	815	[90]
NCT00036816	2002	Terminated	MART-1 NA17-A gp100 tyrosinase peptide	Melanoma	13	[91]
NCT00094653	2004	Completed	gp100	Melanoma	1783	[92]
NCT00454168	2005	Unknown	PR1	Leukemia	244	[93]
NCT00425360	2006	Completed	GV1001	Pancreatic Cancer	1110	[94]
NCT01479244	2011	Completed	NeuVax™	Breast Cancer	758	[95]
NCT01579188	2012	Unknown	GV1001	Non-small Cell Lung Cancer	600	[96]
NCT02654587	2016	Terminated	OSE2101	Non-small Cell Lung Cancer	219	[97]
NCT06472245	2024	Not yet recruiting	OSE2101	Non-small Cell Lung Cancer	363	[98]

**Table 2:** Common CPPs from various sources.

Classification	Name	Sequence	Origin	Characteristics	Reference
Natural					
	TAT48-60	GRKKRRQRRRPQ	Tat protein from HIV-1 virus	Cationic	[162]
	Penetratin	RQIKIWFQNRRMKWKK	Antennapedia Drosophila, bee venom	Cationic	[163]
	Maurocalcine	GDCLPHLKLCKENKDCCS KKCKRRGTNIEKRCR	Scorpio Maurus Palmatus, scorpion venom	Cationic	[164]
	p28	LSTAADMQGWTDGM ASGLDKDYLKPDD	Bacterial protein azurin	Amphiphilic	[165]
Synthetic					
	Pep-1	KETWWETWWT EWSQPKKKRKV	Pep-1, tryptophan dense NLS of Simian antigen	Amphiphilic	[166]
	MAP	KLALKLALK ALKAALKLA	-	Amphiphilic	[167]
	CADY	GLWRALWRLL RSLWRLLWRA	ppTG11 derivative	Amphiphilic	[168]
	Polyarginine	Rn	-	Cationic	[169]
Chimeric					
	pVEC	LLILRRRIRKQAHAHSK	pVEC, Murine Vascular endothelial cadherin tissue	Amphiphilic	[170]
	Transportan	GWTLNSAGYLLGK INLKALAALAKKIL	Galanin–Mastoparan chimeric peptide	Amphiphilic	[171]

**Table 3:** Representative self-assembling peptides related to anti-tumor immunotherapy.

Classification	Name	Immunotherapy Strategy	Type of Cancer	Reference
Nanoparticles				
	T7-PEG-TMC-TBC	Delivery of siRNA-PD-L1 to block inhibitory signals in responsive T cells	Breast Cancer	[205]
	^super^PDL1^EXO^	Activate tumor-specific cytotoxic T lymphocyte (CTL)-related immune responses	Colon Cancer	[208]
	C16-K(PpIX)-PEG8-KDEVD-1MT	Delivery of immune checkpoint inhibitor (1MT) and activate CD8^+^T cells	Colon Cancer	[209]
	MNAs	Stimulate NK cell activity, promote cytokine secretion and cytotoxicity	Hematological Tumors	[210]
	PD-NPs	Induce immunogenic cell death (ICD), block PD-L1	Breast Cancer	[211]
	-	Use monomethyl auristatin E (MMAE) combined with radiotherapy for trimodal precision chemo–radio-immunotherapy	Head and Neck Cancer, Lung Cancer, Colon Cancer	[212]
	AmpFY9	Radiotherapy induces ICD cascade reactions in tumor cells, inhibit IDO-1 to deplete tryptophan and reverse the immunosuppressive tumor microenvironment	Breast Cancer	[213]
	RCPAs	Induce M2-Mφ repolarization to M1 macrophages, enhance macrophage anti-tumor activity and immune regulatory function	Breast Cancer	[214]
Nanofibers				
	SAAPDC	Inhibit MMP, suppress tumor growth, prevent lung metastasis	Liver Cancer	[206]
	LMY1	Block CD47/SIRPα interaction, promote M1-type TAM activation and induce macrophage phagocytosis of tumor cells	Lung Cancer	[215]
	Coil-29	Induce antibody-dependent cellular cytotoxicity (ADCC) and antibody-dependent cellular phagocytosis (ADCP), block immune checkpoints	Melanoma	[216]
	-	Target immune checkpoint CD47, activate adaptive immune system	Colon Cancer	[217]
	octa PEG-PD1-PDL1	Bridge T cells and cancer cells to enhance T cell-mediated cytotoxicity against cancer cells	Colon Cancer	[218]
Hydrogels				
	RADA16	DC recruitment and activation	Unclear	[207]
	L-NIL-MDP	Inhibit inducible nitric oxide synthase (iNOS), deliver immunostimulatory cyclic dinucleotides (CDN)	Oral Cancer	[219]
	PVAX	Block PD-L1, promote DC maturation, induce cytotoxic T lymphocyte infiltration, inhibit tumor recurrence and metastasis	Breast Cancer	[220]
	mPEG-b-PELG	Deliver IL-15, induce recovery of CD8^+^T cells and NK cells to reduce immunosuppression	Melanoma	[221]
	STINGel	Activate STING pathway, enhance innate immunity	Head and Neck Cancer	[222]

## Data Availability

The data of this study are available from the corresponding author on reasonable request.

## References

[1] Siegel R.L., Giaquinto A.N., Jemal A. (2024). Cancer statistics, 2024. CA Cancer J. Clin..

[2] Bray F., Laversanne M., Sung H., Ferlay J., Siegel R.L., Soerjomataram I., Jemal A. (2024). Global cancer statistics 2022: GLOBOCAN estimates of incidence and mortality worldwide for 36 cancers in 185 countries. CA Cancer J. Clin..

[3] Kocarnik J.M., Compton K., Dean F.E., Fu W., Gaw B.L., Harvey J.D., Henrikson H.J., Lu D., Pennini A., Xu R. (2022). Cancer Incidence, Mortality, Years of Life Lost, Years Lived with Disability, and Disability-Adjusted Life Years for 29 Cancer Groups From 2010 to 2019: A Systematic Analysis for the Global Burden of Disease Study 2019. JAMA Oncol..

[4] Sharma P., Goswami S., Raychaudhuri D., Siddiqui B.A., Singh P., Nagarajan A., Liu J., Subudhi S.K., Poon C., Gant K.L. (2023). Immune checkpoint therapy-current perspectives and future directions. Cell.

[5] Albelda S.M. (2024). CAR T cell therapy for patients with solid tumours: Key lessons to learn and unlearn. Nat. Rev. Clin. Oncol..

[6] Tsimberidou A.M., Fountzilas E., Nikanjam M., Kurzrock R. (2020). Review of precision cancer medicine: Evolution of the treatment paradigm. Cancer Treat. Rev..

[7] Grivennikov S.I., Greten F.R., Karin M. (2010). Immunity, inflammation, and cancer. Cell.

[8] Falcone N., Ermis M., Tamay D.G., Mecwan M., Monirizad M., Mathes T.G., Jucaud V., Choroomi A., de Barros N.R., Zhu Y. (2023). Peptide Hydrogels as Immunomaterials and Their Use in Cancer Immunotherapy Delivery. Adv. Healthc. Mater..

[9] Cui J.W., Li Y., Yang Y., Yang H.K., Dong J.M., Xiao Z.H., He X., Guo J.H., Wang R.Q., Dai B. (2024). Tumor immunotherapy resistance: Revealing the mechanism of PD-1 / PD-L1-mediated tumor immune escape. Biomed. Pharmacother..

[10] Havel J.J., Chowell D., Chan T.A. (2019). The evolving landscape of biomarkers for checkpoint inhibitor immunotherapy. Nat. Rev. Cancer.

[11] Kong J., Ha D., Lee J., Kim I., Park M., Im S.H., Shin K., Kim S. (2022). Network-based machine learning approach to predict immunotherapy response in cancer patients. Nat. Commun..

[12] Chen Z., Yue Z., Wang R., Yang K., Li S. (2022). Nanomaterials: A powerful tool for tumor immunotherapy. Front. Immunol..

[13] Harini K., Girigoswami K., Thirumalai A., Girigoswami A. (2024). Polymer-Based Antimicrobial Peptide Mimetics for Treating Multi-drug Resistant Infections: Therapy and Toxicity Evaluation. Int. J. Pept. Res. Ther..

[14] Chen X., Zhao Z., Laster K.V., Liu K., Dong Z. (2024). Advancements in therapeutic peptides: Shaping the future of cancer treatment. Biochim. Biophys. Acta Rev. Cancer.

[15] Zhang L., Huang Y., Lindstrom A.R., Lin T.Y., Lam K.S., Li Y. (2019). Peptide-based materials for cancer immunotherapy. Theranostics.

[16] Sun H., Dong Y., Feijen J., Zhong Z. (2018). Peptide-decorated polymeric nanomedicines for precision cancer therapy. J. Control. Release.

[17] Li B., Niu H., Zhao X., Huang X., Ding Y., Dang K., Yang T., Chen Y., Ma J., Liu X. (2024). Targeted anti-cancer therapy: Co-delivery of VEGF siRNA and Phenethyl isothiocyanate (PEITC) via cRGD-modified lipid nanoparticles for enhanced anti-angiogenic efficacy. Asian J. Pharm. Sci..

[18] Song L., Jiang S., Yang Q., Huang W., Qiu Y., Chen Z., Sun X., Wang T., Wu S., Chen Y. (2024). Development of a Novel Peptide-Based PET Tracer [(68)Ga]Ga-DOTA-BP1 for BCMA Detection in Multiple Myeloma. J. Med. Chem..

[19] Liu B., Zhou H., Tan L., Siu K.T.H., Guan X.Y. (2024). Exploring treatment options in cancer: Tumor treatment strategies. Signal Transduct. Target Ther..

[20] Rahat M.A. (2019). Targeting Angiogenesis with Peptide Vaccines. Front. Immunol..

[21] Liu Q., Wu P., Lei J., Bai P., Zhong P., Yang M., Wei P. (2024). Old concepts, new tricks: How peptide vaccines are reshaping cancer immunotherapy?. Int. J. Biol. Macromol..

[22] Liu D., Liu L., Li X., Wang S., Wu G., Che X. (2024). Advancements and Challenges in Peptide-Based Cancer Vaccination: A Multidisciplinary Perspective. Vaccines.

[23] Zhu Y.S., Tang K., Lv J. (2021). Peptide-drug conjugate-based novel molecular drug delivery system in cancer. Trends Pharmacol. Sci..

[24] Sood A., Jothiswaran V.V., Singh A., Sharma A. (2024). Anticancer peptides as novel immunomodulatory therapeutic candidates for cancer treatment. Explor. Target. Antitumor Ther..

[25] Song Y., Lei L., Cai X., Wei H., Yu C.Y. (2024). Immunomodulatory Peptides for Tumor Treatment. Adv. Healthc. Mater..

[26] Stephens A.J., Burgess-Brown N.A., Jiang S. (2021). Beyond Just Peptide Antigens: The Complex World of Peptide-Based Cancer Vaccines. Front. Immunol..

[27] Bojarska J., Wolf W.M. (2024). Short Peptides as Powerful Arsenal for Smart Fighting Cancer. Cancers.

[28] Sui X., Niu X., Zhou X., Gao Y. (2023). Peptide drugs: A new direction in cancer immunotherapy. Cancer Biol. Med..

[29] Li M., Zhao X., Dai J., Yu Z. (2019). Peptide therapeutics and assemblies for cancer immunotherapy. Sci. China Mater..

[30] Zahedipour F., Jamialahmadi K., Zamani P., Reza Jaafari M. (2023). Improving the efficacy of peptide vaccines in cancer immunotherapy. Int. Immunopharmacol..

[31] Kumai T., Kobayashi H., Harabuchi Y., Celis E. (2017). Peptide vaccines in cancer-old concept revisited. Curr. Opin. Immunol..

[32] van der Bruggen P., Traversari C., Chomez P., Lurquin C., De Plaen E., Van den Eynde B.J., Knuth A., Boon T. (2007). A gene encoding an antigen recognized by cytolytic T lymphocytes on a human melanoma. J. Immunol..

[33] Gardner T.A., Elzey B.D., Hahn N.M. (2012). Sipuleucel-T (Provenge) autologous vaccine approved for treatment of men with asymptomatic or minimally symptomatic castrate-resistant metastatic prostate cancer. Hum. Vaccin. Immunother..

[34] Oh D.Y., Bang Y.J. (2020). HER2-targeted therapies—A role beyond breast cancer. Nat. Rev. Clin. Oncol..

[35] Scholfield D.P., Simms M.S., Bishop M.C. (2003). MUC1 mucin in urological malignancy. BJU Int..

[36] Salmaninejad A., Zamani M.R., Pourvahedi M., Golchehre Z., Hosseini Bereshneh A., Rezaei N. (2016). Cancer/Testis Antigens: Expression, Regulation, Tumor Invasion, and Use in Immunotherapy of Cancers. Immunol. Investig..

[37] Guo N., Niu Z., Yan Z., Liu W., Shi L., Li C., Yao Y., Shi L. (2024). Immunoinformatics Design and In Vivo Immunogenicity Evaluation of a Conserved CTL Multi-Epitope Vaccine Targeting HPV16 E5, E6, and E7 Proteins. Vaccines.

[38] Lee H.H., Hong S.H., Rhee J.H., Lee S.E. (2022). Optimal long peptide for flagellin-adjuvanted HPV E7 cancer vaccine to enhance tumor suppression in combination with anti-PD-1. Transl. Cancer Res..

[39] Lorentzen C.L., Martinenaite E., Kjeldsen J.W., Holmstroem R.B., Mørk S.K., Pedersen A.W., Ehrnrooth E., Andersen M.H., Svane I.M. (2022). Arginase-1 targeting peptide vaccine in patients with metastatic solid tumors—A phase I trial. Front. Immunol..

[40] Kono M., Wakisaka R., Komatsuda H., Hayashi R., Kumai T., Yamaki H., Sato R., Nagato T., Ohkuri T., Kosaka A. (2024). Immunotherapy targeting tumor-associated antigen in a mouse model of head and neck cancer. Head Neck.

[41] Li F., Wu H., Du X., Sun Y., Rausseo B.N., Talukder A., Katailiha A., Elzohary L., Wang Y., Wang Z. (2023). Epidermal Growth Factor Receptor-Targeted Neoantigen Peptide Vaccination for the Treatment of Non-Small Cell Lung Cancer and Glioblastoma. Vaccines.

[42] Liang C., Geng L., Dong Y., Zhang H. (2024). VEGF165b mutant can be used as a protein carrier to form a chimeric tumor vaccine with Mucin1 peptide to elicit an anti-tumor response. Mol. Immunol..

[43] Yang G., Zhou D., Dai Y., Li Y., Wu J., Liu Q., Deng X. (2022). Construction of PEI-EGFR-PD-L1-siRNA dual functional nano-vaccine and therapeutic efficacy evaluation for lung cancer. Thorac. Cancer.

[44] Martinenaite E., Mortensen R.E.J., Hansen M., Orebo Holmström M., Munir Ahmad S., Grønne Dahlager Jørgensen N., Met Ö., Donia M., Svane I.M., Andersen M.H. (2018). Frequent adaptive immune responses against arginase-1. Oncoimmunology.

[45] Gottschalk S., Yu F., Ji M., Kakarla S., Song X.T. (2013). A vaccine that co-targets tumor cells and cancer associated fibroblasts results in enhanced antitumor activity by inducing antigen spreading. PLoS ONE.

[46] Xi X., Ye T., Wang S., Na X., Wang J., Qing S., Gao X., Wang C., Li F., Wei W. (2020). Self-healing microcapsules synergetically modulate immunization microenvironments for potent cancer vaccination. Sci. Adv..

[47] Yan Z., Wu Y., Du J., Li G., Wang S., Cao W., Zhou X., Wu C., Zhang D., Jing X. (2016). A novel peptide targeting Clec9a on dendritic cell for cancer immunotherapy. Oncotarget.

[48] Vivier E., Tomasello E., Baratin M., Walzer T., Ugolini S. (2008). Functions of natural killer cells. Nat. Immunol..

[49] Russick J., Torset C., Hemery E., Cremer I. (2020). NK cells in the tumor microenvironment: Prognostic and theranostic impact. Recent advances and trends. Semin. Immunol..

[50] Tran T.A., Kim Y.H., Duong T.H., Jung S., Kim I.Y., Moon K.S., Jang W.Y., Lee H.J., Lee J.J., Jung T.Y. (2020). Peptide Vaccine Combined Adjuvants Modulate Anti-tumor Effects of Radiation in Glioblastoma Mouse Model. Front. Immunol..

[51] Chapel A., Garcia-Beltran W.F., Hölzemer A., Ziegler M., Lunemann S., Martrus G., Altfeld M. (2017). Peptide-specific engagement of the activating NK cell receptor KIR2DS1. Sci. Rep..

[52] Yokota C., Nakata J., Takano K., Nakajima H., Hayashibara H., Minagawa H., Chiba Y., Hirayama R., Kijima N., Kinoshita M. (2021). Distinct difference in tumor-infiltrating immune cells between Wilms’ tumor gene 1 peptide vaccine and anti-programmed cell death-1 antibody therapies. Neurooncol. Adv..

[53] Shapouri-Moghaddam A., Mohammadian S., Vazini H., Taghadosi M., Esmaeili S.A., Mardani F., Seifi B., Mohammadi A., Afshari J.T., Sahebkar A. (2018). Macrophage plasticity, polarization, and function in health and disease. J. Cell. Physiol..

[54] Pan Y., Yu Y., Wang X., Zhang T. (2020). Tumor-Associated Macrophages in Tumor Immunity. Front. Immunol..

[55] Qian Y., Qiao S., Dai Y., Xu G., Dai B., Lu L., Yu X., Luo Q., Zhang Z. (2017). Molecular-Targeted Immunotherapeutic Strategy for Melanoma via Dual-Targeting Nanoparticles Delivering Small Interfering RNA to Tumor-Associated Macrophages. ACS Nano.

[56] Ngambenjawong C., Cieslewicz M., Schellinger J.G., Pun S.H. (2016). Synthesis and evaluation of multivalent M2pep peptides for targeting alternatively activated M2 macrophages. J. Control. Release.

[57] Patel S., Fu S., Mastio J., Dominguez G.A., Purohit A., Kossenkov A., Lin C., Alicea-Torres K., Sehgal M., Nefedova Y. (2018). Unique pattern of neutrophil migration and function during tumor progression. Nat. Immunol..

[58] Smith J.A. (1994). Neutrophils, host defense, and inflammation: A double-edged sword. J. Leukoc. Biol..

[59] Feola S., Hamdan F., Russo S., Chiaro J., Fusciello M., Feodoroff M., Antignani G., D’Alessio F., Mölsä R., Stigzelius V. (2024). Novel peptide-based oncolytic vaccine for enhancement of adaptive antitumor immune response via co-engagement of innate Fcγ and Fcα receptors. J. Immunother. Cancer.

[60] Miettinen H.M., Gripentrog J.M., Lord C.I., Nagy J.O. (2018). CD177-mediated nanoparticle targeting of human and mouse neutrophils. PLoS ONE.

[61] Mellman I., Coukos G., Dranoff G. (2011). Cancer immunotherapy comes of age. Nature.

[62] Cen L., Zhang Z., Sun Y., Wu N., Shao J., Qian Z., Tian M., Ke Y., Liu B. (2024). Efficacy of MAGE-A4 long peptide as a universal immunoprevention cancer vaccine. Cancer Cell Int..

[63] Zeng L., Zheng W., Zhang J., Wang J., Ji Q., Wu X., Meng Y., Zhu X. (2023). An epitope encoded by uORF of RNF10 elicits a therapeutic anti-tumor immune response. Mol. Ther. Oncolytics.

[64] Nishikawa H., Sakaguchi S. (2010). Regulatory T cells in tumor immunity. Int. J. Cancer.

[65] Zou W. (2006). Regulatory T cells, tumour immunity and immunotherapy. Nat. Rev. Immunol..

[66] Lozano T., Gorraiz M., Lasarte-Cía A., Ruiz M., Rabal O., Oyarzabal J., Hervás-Stubbs S., Llopiz D., Sarobe P., Prieto J. (2017). Blockage of FOXP3 transcription factor dimerization and FOXP3/AML1 interaction inhibits T regulatory cell activity: Sequence optimization of a peptide inhibitor. Oncotarget.

[67] Wang S., Jiang S., Li X., Huang H., Qiu X., Yu M., Yang X., Liu F., Wang C., Shen W. (2024). FGL2(172-220) peptides improve the antitumor effect of HCMV-IE1mut vaccine against glioblastoma by modulating immunosuppressive cells in the tumor microenvironment. Oncoimmunology.

[68] Yuen G.J., Demissie E., Pillai S. (2016). B lymphocytes and cancer: A love-hate relationship. Trends Cancer.

[69] Kaumaya P.T. (2015). A paradigm shift: Cancer therapy with peptide-based B-cell epitopes and peptide immunotherapeutics targeting multiple solid tumor types: Emerging concepts and validation of combination immunotherapy. Hum. Vaccin. Immunother..

[70] Zhou Y., Li X., Guo Y., Wu Y., Yin L., Tu L., Hong S., Cai H., Ding F. (2024). Synthetic self-adjuvanted multivalent Mucin 1 (MUC1) glycopeptide vaccines with improved in vivo antitumor efficacy. MedComm.

[71] Zhou S.H., Zhang R.Y., Wen Y., Zou Y.K., Ding D., Bian M.M., Cui H.Y., Guo J. (2024). Multifunctional Lipidated Protein Carrier with a Built-In Adjuvant as a Universal Vaccine Platform Potently Elevates Immunogenicity of Weak Antigens. J. Med. Chem..

[72] Zhang R.Y., Wen Y., He C.B., Zhou S.H., Wu Y.H., Wang E.Y., Feng R.R., Ding D., Du J.J., Gao X.F. (2024). Conjugation of TLR7 and TLR7/8 agonists onto weak protein antigen via versatile oxime ligation for enhanced vaccine efficacy. Int. J. Biol. Macromol..

[73] Du J.J., Wang C.W., Xu W.B., Zhang L., Tang Y.K., Zhou S.H., Gao X.F., Yang G.F., Guo J. (2020). Multifunctional Protein Conjugates with Built-in Adjuvant (Adjuvant-Protein-Antigen) as Cancer Vaccines Boost Potent Immune Responses. iScience.

[74] Vincent C.A., Remeseiro S. (2024). The immune response behind peptide vaccination in diffuse midline glioma. Mol. Oncol..

[75] Johanns T.M., Garfinkle E.A.R., Miller K.E., Livingstone A.J., Roberts K.F., Rao Venkata L.P., Dowling J.L., Chicoine M.R., Dacey R.G., Zipfel G.J. (2024). Integrating Multisector Molecular Characterization into Personalized Peptide Vaccine Design for Patients with Newly Diagnosed Glioblastoma. Clin. Cancer Res..

[76] Ahluwalia M.S., Reardon D.A., Abad A.P., Curry W.T., Wong E.T., Figel S.A., Mechtler L.L., Peereboom D.M., Hutson A.D., Withers H.G. (2023). Phase IIa Study of SurVaxM Plus Adjuvant Temozolomide for Newly Diagnosed Glioblastoma. J. Clin. Oncol..

[77] Schwartzentruber D.J., Lawson D.H., Richards J.M., Conry R.M., Miller D.M., Treisman J., Gailani F., Riley L., Conlon K., Pockaj B. (2011). gp100 peptide vaccine and interleukin-2 in patients with advanced melanoma. N. Engl. J. Med..

[78] Hodi F.S., O’Day S.J., McDermott D.F., Weber R.W., Sosman J.A., Haanen J.B., Gonzalez R., Robert C., Schadendorf D., Hassel J.C. (2010). Improved survival with ipilimumab in patients with metastatic melanoma. N. Engl. J. Med..

[79] Brentville V.A., Symonds P., Chua J., Skinner A., Daniels I., Cook K.W., Koncarevic S., Martinez-Pinna R., Shah S., Choudhury R.H. (2022). Citrullinated glucose-regulated protein 78 is a candidate target for melanoma immunotherapy. Front. Immunol..

[80] Schoen R.E., Boardman L.A., Cruz-Correa M., Bansal A., Kastenberg D., Hur C., Dzubinski L., Kaufman S.F., Rodriguez L.M., Richmond E. (2023). Randomized, Double-Blind, Placebo-Controlled Trial of MUC1 Peptide Vaccine for Prevention of Recurrent Colorectal Adenoma. Clin. Cancer Res..

[81] Correale P., Botta C., Martino E.C., Ulivieri C., Battaglia G., Carfagno T., Rossetti M.G., Fioravanti A., Guidelli G.M., Cheleschi S. (2016). Phase Ib study of poly-epitope peptide vaccination to thymidylate synthase (TSPP) and GOLFIG chemo-immunotherapy for treatment of metastatic colorectal cancer patients. Oncoimmunology.

[82] Taniguchi H., Iwasa S., Yamazaki K., Yoshino T., Kiryu C., Naka Y., Liew E.L., Sakata Y. (2017). Phase 1 study of OCV-C02, a peptide vaccine consisting of two peptide epitopes for refractory metastatic colorectal cancer. Cancer Sci..

[83] Adotévi O., Vernerey D., Jacoulet P., Meurisse A., Laheurte C., Almotlak H., Jacquin M., Kaulek V., Boullerot L., Malfroy M. (2023). Safety, Immunogenicity, and 1-Year Efficacy of Universal Cancer Peptide-Based Vaccine in Patients with Refractory Advanced Non-Small-Cell Lung Cancer: A Phase Ib/Phase IIa De-Escalation Study. J. Clin. Oncol..

[84] Kjeldsen J.W., Iversen T.Z., Engell-Noerregaard L., Mellemgaard A., Andersen M.H., Svane I.M. (2018). Durable Clinical Responses and Long-Term Follow-Up of Stage III-IV Non-Small-Cell Lung Cancer (NSCLC) Patients Treated with IDO Peptide Vaccine in a Phase I Study-A Brief Research Report. Front. Immunol..

[85] Koido S., Taguchi J., Shimabuku M., Kan S., Bito T., Misawa T., Ito Z., Uchiyama K., Saruta M., Tsukinaga S. (2024). Dendritic cells pulsed with multifunctional Wilms’ tumor 1 (WT1) peptides combined with multiagent chemotherapy modulate the tumor microenvironment and enable conversion surgery in pancreatic cancer. J. Immunother. Cancer.

[86] Manning-Geist B.L., Gnjatic S., Aghajanian C., Konner J., Kim S.H., Sarasohn D., Soldan K., Tew W.P., Sarlis N.J., Zamarin D. (2023). Phase I Study of a Multivalent WT1 Peptide Vaccine (Galinpepimut-S) in Combination with Nivolumab in Patients with WT1-Expressing Ovarian Cancer in Second or Third Remission. Cancers.

[87] O’Shea A.E., Clifton G.T., Qiao N., Heckman-Stoddard B.M., Wojtowicz M., Dimond E., Bedrosian I., Weber D., Garber J.E., Husband A. (2023). Phase II Trial of Nelipepimut-S Peptide Vaccine in Women with Ductal Carcinoma In Situ. Cancer Prev. Res..

[88] You Z., Zhou W., Weng J., Feng H., Liang P., Li Y., Shi F. (2021). Application of HER2 peptide vaccines in patients with breast cancer: A systematic review and meta-analysis. Cancer Cell Int..

[89] A Phase III Multi-Institutional Randomized Study of Immunization With the gp100: 209-217 (210M) Peptide Followed by High Dose IL-2 vs. High Dose IL-2 Alone in Patients with Metastatic Melanoma. https://clinicaltrials.gov/study/NCT00019682.

[90] A Randomized, Placebo-Controlled Phase III Trial of Yeast Derived GM-CSF Versus Peptide Vaccination Versus GM-CSF Plus Peptide Vaccination Versus Placebo in Patients With “No Evidence of Disease” After Complete Surgical Resection of “Locally Advanced” and/or Stage IV Melanoma. https://clinicaltrials.gov/study/NCT01989572.

[91] Randomized Phase III Study of Adjuvant Immunization with The NA17.A2 and Melanoma Differentiation Peptites In HLA-A2 Patients with Primary Ocular Melanoma at High Risk of Relapse. https://clinicaltrials.gov/study/NCT00036816.

[92] A Randomized, Double-Blind, Multicenter Study Comparing MDX-010 Monotherapy, MDX-010 in Combination with a Melanoma Peptide Vaccine, and Melanoma Vaccine Monotherapy in HLA-A2*0201-Positive Patients with Previously Treated Unresectable Stage III or IV Melanoma. https://clinicaltrials.gov/study/NCT00094653.

[93] A Phase 3, Randomized, Double-Blind, Multicenter Study of Proteinase 3 PR1 Peptide Mixed with Montanide ISA-51 VG Adjuvant and Administered with GM-CSF in Elderly Patients with AML in First Complete Remission or Adults in Second Complete Remission: A Pivotal Study. https://clinicaltrials.gov/study/NCT00454168.

[94] A Prospective, Phase III, Controlled, Multicentre, Randomised Clinical Trial Comparing Combination Gemcitabine and Capecitabine Therapy with Concurrent and Sequential Chemoimmunotherapy Using a Telomerase Vaccine in Locally Advanced and Metastatic Pancreatic Cancer [TELOVAC]. https://clinicaltrials.gov/study/NCT00425360.

[95] PRESENT: Prevention of Recurrence in Early-Stage, Node-Positive Breast Cancer with Low to Intermediate HER2 Expressions with NeuVax™Treatment. https://clinicaltrials.gov/study/NCT01479244.

[96] A Double-blind, Multicenter, Randomized Phase III Study of the Telomerase Vaccine, GV1001 Administered After Curative Intent Chemo-radiotherapy in Patients with Inoperable Stage III Non-small Cell Lung Cancer Compared to Best Supportive Care. https://clinicaltrials.gov/study/NCT01579188.

[97] A Randomized Phase III Trial of OSE2101 Compared with Chemotherapy (Docetaxel or Pemetrexed) in HLA-A2 Positive Patients with Advanced Non-Small Cell Lung Cancer with Progressive Disease After Immune Checkpoint Inhibitors. https://clinicaltrials.gov/study/NCT02654587.

[98] A Randomised, Open-label, Phase 3 Trial Comparing the Efficacy and Safety of OSE2101 Versus Docetaxel in HLA-A2 Positive Patients with Metastatic Non-Small Cell Lung Cancer (NSCLC) With Secondary Resistance to Immune Checkpoint Inhibitor. https://clinicaltrials.gov/study/NCT06472245.

[99] Oji Y., Inoue M., Takeda Y., Hosen N., Shintani Y., Kawakami M., Harada T., Murakami Y., Iwai M., Fukuda M. (2018). WT1 peptide-based immunotherapy for advanced thymic epithelial malignancies. Int. J. Cancer.

[100] Buteau C., Markovic S.N., Celis E. (2002). Challenges in the development of effective peptide vaccines for cancer. Mayo Clin. Proc..

[101] Negahdaripour M., Nezafat N., Hajighahramani N., Rahmatabadi S.S., Ghasemi Y. (2017). Investigating CRISPR-Cas systems in Clostridium botulinum via bioinformatics tools. Infect. Genet. Evol..

[102] Marincola F.M., Jaffee E.M., Hicklin D.J., Ferrone S. (2000). Escape of human solid tumors from T-cell recognition: Molecular mechanisms and functional significance. Adv. Immunol..

[103] McGranahan N., Swanton C. (2017). Clonal Heterogeneity and Tumor Evolution: Past, Present, and the Future. Cell.

[104] Betts G., Jones E., Junaid S., El-Shanawany T., Scurr M., Mizen P., Kumar M., Jones S., Rees B., Williams G. (2012). Suppression of tumour-specific CD4⁺ T cells by regulatory T cells is associated with progression of human colorectal cancer. Gut.

[105] Xia A., Zhang Y., Xu J., Yin T., Lu X.J. (2019). T Cell Dysfunction in Cancer Immunity and Immunotherapy. Front. Immunol..

[106] Melero I., Rouzaut A., Motz G.T., Coukos G. (2014). T-cell and NK-cell infiltration into solid tumors: A key limiting factor for efficacious cancer immunotherapy. Cancer Discov..

[107] Hatziioannou A., Alissafi T., Verginis P. (2017). Myeloid-derived suppressor cells and T regulatory cells in tumors: Unraveling the dark side of the force. J. Leukoc. Biol..

[108] Han B.S., Ji S., Woo S., Lee J.H., Sin J.I. (2019). Regulation of the translation activity of antigen-specific mRNA is responsible for antigen loss and tumor immune escape in a HER2-expressing tumor model. Sci. Rep..

[109] Khong H., Overwijk W.W. (2016). Adjuvants for peptide-based cancer vaccines. J. Immunother. Cancer.

[110] Gouttefangeas C., Rammensee H.G. (2018). Personalized cancer vaccines: Adjuvants are important, too. Cancer Immunol. Immunother..

[111] Rajapakse M., Schmidt B., Feng L., Brusic V. (2007). Predicting peptides binding to MHC class II molecules using multi-objective evolutionary algorithms. BMC Bioinform..

[112] Tung C.W., Ho S.Y. (2007). POPI: Predicting immunogenicity of MHC class I binding peptides by mining informative physicochemical properties. Bioinformatics.

[113] Haj A.K., Breitbach M.E., Baker D.A., Mohns M.S., Moreno G.K., Wilson N.A., Lyamichev V., Patel J., Weisgrau K.L., Dudley D.M. (2020). High-Throughput Identification of MHC Class I Binding Peptides Using an Ultradense Peptide Array. J. Immunol..

[114] Lu Y.C., Zheng Z., Robbins P.F., Tran E., Prickett T.D., Gartner J.J., Li Y.F., Ray S., Franco Z., Bliskovsky V. (2018). An Efficient Single-Cell RNA-Seq Approach to Identify Neoantigen-Specific T Cell Receptors. Mol. Ther..

[115] Iinuma H., Fukushima R., Inaba T., Tamura J., Inoue T., Ogawa E., Horikawa M., Ikeda Y., Matsutani N., Takeda K. (2014). Phase I clinical study of multiple epitope peptide vaccine combined with chemoradiation therapy in esophageal cancer patients. J. Transl. Med..

[116] Lilleby W., Seierstad T., Inderberg E.M., Hole K.H. (2023). Impact of human telomerase reverse transcriptase peptide vaccine combined with androgen deprivation therapy and radiotherapy in de novo metastatic prostate cancer: Long-term clinical monitoring. Int. J. Cancer.

[117] Slingluff C.L., Petroni G.R., Chianese-Bullock K.A., Smolkin M.E., Ross M.I., Haas N.B., von Mehren M., Grosh W.W. (2011). Randomized multicenter trial of the effects of melanoma-associated helper peptides and cyclophosphamide on the immunogenicity of a multipeptide melanoma vaccine. J. Clin. Oncol..

[118] Jeon D., Hill E., Moseman J.E., McNeel D.G. (2024). Combining toll-like receptor agonists with immune checkpoint blockade affects antitumor vaccine efficacy. J. Immunother. Cancer.

[119] Desai R., Suryadevara C.M., Batich K.A., Farber S.H., Sanchez-Perez L., Sampson J.H. (2016). Emerging immunotherapies for glioblastoma. Expert Opin. Emerg. Drugs.

[120] Nishiyama H., Yonese J., Kawahara T., Matsumoto R., Miyake H., Matsubara N., Uemura H., Eto M., Azuma H., Obara W. (2024). TAS0313 plus Pembrolizumab for Post-Chemotherapy Immune Checkpoint Inhibitor-Naïve Locally Advanced or Metastatic Urothelial Carcinoma. Mol. Cancer Ther..

[121] Reddy S.T., Swartz M.A., Hubbell J.A. (2006). Targeting dendritic cells with biomaterials: Developing the next generation of vaccines. Trends Immunol..

[122] Wang J., Hu X., Xiang D. (2018). Nanoparticle drug delivery systems: An excellent carrier for tumor peptide vaccines. Drug Deliv..

[123] Marrack P., McKee A.S., Munks M.W. (2009). Towards an understanding of the adjuvant action of aluminium. Nat. Rev. Immunol..

[124] Reed S.G., Orr M.T., Fox C.B. (2013). Key roles of adjuvants in modern vaccines. Nat. Med..

[125] Dehghankhold M., Nezafat N., Farahmandnejad M., Abolmaali S.S., Tamaddon A.M. (2024). Immunoinformatic approach to design an efficient multi-epitope peptide vaccine against melanoma. Biotechnol. Appl. Biochem..

[126] Li X., Wang S., Zhu X., Zhangsun D., Wu Y., Luo S. (2020). Effects of Cyclization on Activity and Stability of α-Conotoxin TxIB. Mar. Drugs.

[127] Lath A., Santal A.R., Kaur N., Kumari P., Singh N.P. (2023). Anti-cancer peptides: Their current trends in the development of peptide-based therapy and anti-tumor drugs. Biotechnol. Genet. Eng. Rev..

[128] Guzmán-Rodríguez J.J., Ochoa-Zarzosa A., López-Gómez R., López-Meza J.E. (2015). Plant antimicrobial peptides as potential anticancer agents. Biomed. Res. Int..

[129] Agrawal S., Acharya D., Adholeya A., Barrow C.J., Deshmukh S.K. (2017). Nonribosomal Peptides from Marine Microbes and Their Antimicrobial and Anticancer Potential. Front. Pharmacol..

[130] Ibrahim O. (2019). Classification of Antimicrobial Peptides Bacteriocins, and the Nature of Some Bacteriocins with Potential Applications in Food Safety and Bio-Pharmaceuticals. EC Microbiol..

[131] Akbarian M., Khani A., Eghbalpour S., Uversky V.N. (2022). Bioactive Peptides: Synthesis, Sources, Applications, and Proposed Mechanisms of Action. Int. J. Mol. Sci..

[132] Orafaie A., Bahrami A.R., Matin M.M. (2021). Use of anticancer peptides as an alternative approach for targeted therapy in breast cancer: A review. Nanomedicine.

[133] Jang J.P., Jung H.J., Han J.M., Jung N., Kim Y., Kwon H.J., Ko S.K., Soung N.K., Jang J.H., Ahn J.S. (2017). Two cyclic hexapeptides from Penicillium sp. FN070315 with antiangiogenic activities. PLoS ONE.

[134] Yi Z.F., Cho S.G., Zhao H., Wu Y.Y., Luo J., Li D., Yi T., Xu X., Wu Z., Liu M. (2009). A novel peptide from human apolipoprotein(a) inhibits angiogenesis and tumor growth by targeting c-Src phosphorylation in VEGF-induced human umbilical endothelial cells. Int. J. Cancer.

[135] Hilchie A.L., Hoskin D.W., Power Coombs M.R. (2019). Anticancer Activities of Natural and Synthetic Peptides. Adv. Exp. Med. Biol..

[136] Diao Y., Han W., Zhao H., Zhu S., Liu X., Feng X., Gu J., Yao C., Liu S., Sun C. (2012). Designed synthetic analogs of the α-helical peptide temporin-La with improved antitumor efficacies via charge modification and incorporation of the integrin αvβ3 homing domain. J. Pept. Sci..

[137] Vermaelen K. (2019). Vaccine Strategies to Improve Anti-cancer Cellular Immune Responses. Front. Immunol..

[138] Conlon J.M., Mechkarska M., Lukic M.L., Flatt P.R. (2014). Potential therapeutic applications of multifunctional host-defense peptides from frog skin as anti-cancer, anti-viral, immunomodulatory, and anti-diabetic agents. Peptides.

[139] Dai J., Dong X., Liu R., Chen B., Dong X., Wang Q., Hu J.J., Xia F., Lou X. (2022). A peptide-AIEgen nanocomposite mediated whole cancer immunity cycle-cascade amplification for improved immunotherapy of tumor. Biomaterials.

[140] Shen W., Shi P., Dong Q., Zhou X., Chen C., Sui X., Tian W., Zhu X., Wang X., Jin S. (2023). Discovery of a novel dual-targeting D-peptide to block CD24/Siglec-10 and PD-1/PD-L1 interaction and synergize with radiotherapy for cancer immunotherapy. J. Immunother. Cancer.

[141] Yang Q., Peng J., Shi K., Xiao Y., Liu Q., Han R., Wei X., Qian Z. (2019). Rationally designed peptide-conjugated gold/platinum nanosystem with active tumor-targeting for enhancing tumor photothermal-immunotherapy. J. Control. Release.

[142] Chen Y., Li W., Wang Z., Yu Y., Li J., Ding Y., Hu Z., Liu Q., Yang Z., Gao J. (2024). A Transformable Supramolecular Bispecific Cell Engager for Augmenting Natural Killer and T Cell-Based Cancer Immunotherapy. Adv. Mater..

[143] Taleb M., Atabakhshi-Kashi M., Wang Y., Rezvani Alanagh H., Farhadi Sabet Z., Li F., Cheng K., Li C., Qi Y., Nie G. (2021). Bifunctional Therapeutic Peptide Assembled Nanoparticles Exerting Improved Activities of Tumor Vessel Normalization and Immune Checkpoint Inhibition. Adv. Healthc. Mater..

[144] Yang X., Li W., Li S., Chen S., Hu Z., He Z., Zhu X., Niu X., Zhou X., Li H. (2024). Fish oil-based microemulsion can efficiently deliver oral peptide blocking PD-1/PD-L1 and simultaneously induce ferroptosis for cancer immunotherapy. J. Control. Release.

[145] Zhang L., Jiang Z., Yang X., Qian Y., Wang M., Wu S., Li L., Jia F., Wang Z., Hu Z. (2023). A Totipotent “All-In-One” Peptide Sequentially Blocks Immune Checkpoint and Reverses the Immunosuppressive Tumor Microenvironment. Adv. Mater..

[146] Xing Y., Peng A., Yang J., Cheng Z., Yue Y., Liu F., Li F., Liu Y., Liu Q. (2024). Precisely Activating cGAS-STING Pathway with a Novel Peptide-Based Nanoagonist to Potentiate Immune Checkpoint Blockade Cancer Immunotherapy. Adv. Sci..

[147] Wang H., Sun Y., Zhou X., Chen C., Jiao L., Li W., Gou S., Li Y., Du J., Chen G. (2020). CD47/SIRPα blocking peptide identification and synergistic effect with irradiation for cancer immunotherapy. J. Immunother. Cancer.

[148] Johnston R.J., Comps-Agrar L., Hackney J., Yu X., Huseni M., Yang Y., Park S., Javinal V., Chiu H., Irving B. (2014). The immunoreceptor TIGIT regulates antitumor and antiviral CD8(+) T cell effector function. Cancer Cell.

[149] Zhou X., Zuo C., Li W., Shi W., Zhou X., Wang H., Chen S., Du J., Chen G., Zhai W. (2020). A Novel d-Peptide Identified by Mirror-Image Phage Display Blocks TIGIT/PVR for Cancer Immunotherapy. Angew. Chem. Int. Ed. Engl..

[150] Tang S., Zhou L., He H., Cui L., Ren Z., Tai Y., Xie Z., Cao Y., Meng D., Liu Q. (2022). MnO(2)-melittin nanoparticles serve as an effective anti-tumor immunotherapy by enhancing systemic immune response. Biomaterials.

[151] Wang D., Liu J., Li T., Wang Y., Liu X., Bai Y., Wang C., Ju S., Huang S., Yang C. (2022). A VEGFR targeting peptide-drug conjugate (PDC) suppresses tumor angiogenesis in a TACE model for hepatocellular carcinoma therapy. Cell Death Discov..

[152] Liu J., Bai Y., Liu X., Zhou B., Sun P., Wang Y., Ju S., Zhou C., Wang C., Yao W. (2024). Enhanced efficacy of combined VEGFR peptide-drug conjugate and anti-PD-1 antibody in treating hepatocellular carcinoma. Sci. Rep..

[153] Kan X., Zhou G., Zhang F., Ji H., Shin D.S., Monsky W., Zheng C., Yang X. (2022). Enhanced efficacy of direct immunochemotherapy for hepatic cancer with image-guided intratumoral radiofrequency hyperthermia. J. Immunother. Cancer.

[154] Bauso L.V., La Fauci V., Munaò S., Bonfiglio D., Armeli A., Maimone N., Longo C., Calabrese G. (2024). Biological Activity of Natural and Synthetic Peptides as Anticancer Agents. Int. J. Mol. Sci..

[155] Bruno B.J., Miller G.D., Lim C.S. (2013). Basics and recent advances in peptide and protein drug delivery. Ther. Deliv..

[156] Alamdari-Palangi V., Jaberi K.R., Shahverdi M., Naeimzadeh Y., Tajbakhsh A., Khajeh S., Razban V., Fallahi J. (2023). Recent advances and applications of peptide-agent conjugates for targeting tumor cells. J. Cancer Res. Clin. Oncol..

[157] Samec T., Boulos J., Gilmore S., Hazelton A., Alexander-Bryant A. (2022). Peptide-based delivery of therapeutics in cancer treatment. Mater. Today Bio.

[158] Bellat V., Ting R., Southard T.L., Vahdat L., Molina H., Fernandez J., Aras O., Stokol T., Law B. (2018). Functional Peptide Nanofibers with Unique Tumor Targeting and Enzyme-Induced Local Retention Properties. Adv. Funct. Mater..

[159] Shi J., Kantoff P.W., Wooster R., Farokhzad O.C. (2017). Cancer nanomedicine: Progress, challenges and opportunities. Nat. Rev. Cancer.

[160] Mukherjee A.G., Wanjari U.R., Gopalakrishnan A.V., Bradu P., Biswas A., Ganesan R., Renu K., Dey A., Vellingiri B., El Allali A. (2023). Evolving strategies and application of proteins and peptide therapeutics in cancer treatment. Biomed. Pharmacother..

[161] Cui C., Huo Q., Xiong X., Na S., Mitsuda M., Minami K., Li B., Yokota H. (2024). P18: Novel Anticancer Peptide from Induced Tumor-Suppressing Cells Targeting Breast Cancer and Bone Metastasis. Cancers.

[162] Mai J.C., Shen H., Watkins S.C., Cheng T., Robbins P.D. (2002). Efficiency of protein transduction is cell type-dependent and is enhanced by dextran sulfate. J. Biol. Chem..

[163] Derossi D., Joliot A.H., Chassaing G., Prochiantz A. (1994). The third helix of the Antennapedia homeodomain translocates through biological membranes. J. Biol. Chem..

[164] Rádis-Baptista G. (2021). Cell-Penetrating Peptides Derived from Animal Venoms and Toxins. Toxins.

[165] Naffouje S.A., Goto M., Coward L.U., Gorman G.S., Christov K., Wang J., Green A., Shilkaitis A., Das Gupta T.K., Yamada T. (2022). Nontoxic Tumor-Targeting Optical Agents for Intraoperative Breast Tumor Imaging. J. Med. Chem..

[166] Morris M.C., Depollier J., Mery J., Heitz F., Divita G. (2001). A peptide carrier for the delivery of biologically active proteins into mammalian cells. Nat. Biotechnol..

[167] Silva S., Kurrikoff K., Langel Ü., Almeida A.J., Vale N. (2022). A Second Life for MAP, a Model Amphipathic Peptide. Int. J. Mol. Sci..

[168] Rittner K., Benavente A., Bompard-Sorlet A., Heitz F., Divita G., Brasseur R., Jacobs E. (2002). New basic membrane-destabilizing peptides for plasmid-based gene delivery in vitro and in vivo. Mol. Ther..

[169] Futaki S. (2005). Membrane-permeable arginine-rich peptides and the translocation mechanisms. Adv. Drug Deliv. Rev..

[170] Kang S., Jeon S., Kim S., Chang Y.K., Kim Y.C. (2020). Development of a pVEC peptide-based ribonucleoprotein (RNP) delivery system for genome editing using CRISPR/Cas9 in Chlamydomonas reinhardtii. Sci. Rep..

[171] Lindgren M., Gallet X., Soomets U., Hällbrink M., Bråkenhielm E., Pooga M., Brasseur R., Langel U. (2000). Translocation properties of novel cell penetrating transportan and penetratin analogues. Bioconjug. Chem..

[172] Allolio C., Magarkar A., Jurkiewicz P., Baxová K., Javanainen M., Mason P.E., Šachl R., Cebecauer M., Hof M., Horinek D. (2018). Arginine-rich cell-penetrating peptides induce membrane multilamellarity and subsequently enter via formation of a fusion pore. Proc. Natl. Acad. Sci. USA.

[173] Gessner I., Klimpel A., Klußmann M., Neundorf I., Mathur S. (2020). Interdependence of charge and secondary structure on cellular uptake of cell penetrating peptide functionalized silica nanoparticles. Nanoscale Adv..

[174] Koo J.H., Kim G.R., Nam K.H., Choi J.M. (2022). Unleashing cell-penetrating peptide applications for immunotherapy. Trends Mol. Med..

[175] Fujioka Y., Ueki H., A R., Sasajima A., Tomono T., Ukawa M., Yagi H., Sakuma S., Kitagawa K., Shirakawa T. (2024). The Improved Antigen Uptake and Presentation of Dendritic Cells Using Cell-Penetrating D-octaarginine-Linked PNVA-co-AA as a Novel Dendritic Cell-Based Vaccine. Int. J. Mol. Sci..

[176] Lim S., Lee J.A., Koo J.H., Kang T.G., Ha S.J., Choi J.M. (2016). Cell Type Preference of a Novel Human Derived Cell-Permeable Peptide dNP2 and TAT in Murine Splenic Immune Cells. PLoS ONE.

[177] Derouazi M., Di Berardino-Besson W., Belnoue E., Hoepner S., Walther R., Benkhoucha M., Teta P., Dufour Y., Yacoub Maroun C., Salazar A.M. (2015). Novel Cell-Penetrating Peptide-Based Vaccine Induces Robust CD4+ and CD8+ T Cell-Mediated Antitumor Immunity. Cancer Res..

[178] Wang R.F., Wang H.Y. (2002). Enhancement of antitumor immunity by prolonging antigen presentation on dendritic cells. Nat. Biotechnol..

[179] Jin Z., Piao L., Sun G., Lv C., Jing Y., Jin R. (2021). Dual functional nanoparticles efficiently across the blood-brain barrier to combat glioblastoma via simultaneously inhibit the PI3K pathway and NKG2A axis. J. Drug Target..

[180] Kim D.H., Park H.J., Lim S., Koo J.H., Lee H.G., Choi J.O., Oh J.H., Ha S.J., Kang M.J., Lee C.M. (2018). Regulation of chitinase-3-like-1 in T cell elicits Th1 and cytotoxic responses to inhibit lung metastasis. Nat. Commun..

[181] Huang J., Wang K., Fu X., Zhu M., Chen X., Gao Y., Ma P., Duan X., Men K. (2023). Efficient Colon Cancer Immunogene Therapy Through Co-Delivery of IL-22BP mRNA and Tumor Cell Lysate by CLSV Nanoparticles. Int. J. Nanomed..

[182] Huang J., Wang K., Wu S., Zhang J., Chen X., Lei S., Wu J., Men K., Duan X. (2024). Tumor Cell Lysate-Based Multifunctional Nanoparticles Facilitate Enhanced mRNA Delivery and Immune Stimulation for Melanoma Gene Therapy. Mol. Pharm..

[183] Amantana A., Moulton H.M., Cate M.L., Reddy M.T., Whitehead T., Hassinger J.N., Youngblood D.S., Iversen P.L. (2007). Pharmacokinetics, biodistribution, stability and toxicity of a cell-penetrating peptide-morpholino oligomer conjugate. Bioconjug. Chem..

[184] David A. (2017). Peptide ligand-modified nanomedicines for targeting cells at the tumor microenvironment. Adv. Drug Deliv. Rev..

[185] Chen Q., Liu L., Lu Y., Chen X., Zhang Y., Zhou W., Guo Q., Li C., Zhang Y., Zhang Y. (2019). Tumor Microenvironment-Triggered Aggregated Magnetic Nanoparticles for Reinforced Image-Guided Immunogenic Chemotherapy. Adv. Sci..

[186] Temming K., Schiffelers R.M., Molema G., Kok R.J. (2005). RGD-based strategies for selective delivery of therapeutics and imaging agents to the tumour vasculature. Drug Resist. Updates.

[187] Schraa A.J., Kok R.J., Botter S.M., Withoff S., Meijer D.K., de Leij L.F., Molema G. (2004). RGD-modified anti-CD3 antibodies redirect cytolytic capacity of cytotoxic T lymphocytes toward alphavbeta3-expressing endothelial cells. Int. J. Cancer.

[188] Gao S., Li T., Guo Y., Sun C., Xianyu B., Xu H. (2020). Selenium-Containing Nanoparticles Combine the NK Cells Mediated Immunotherapy with Radiotherapy and Chemotherapy. Adv. Mater..

[189] Chakraborty K., Mondal J., An J.M., Park J., Lee Y.K. (2023). Advances in Radionuclides and Radiolabelled Peptides for Cancer Therapeutics. Pharmaceutics.

[190] Yu S.S., Athreya K., Liu S.V., Schally A.V., Tsao-Wei D., Groshen S., Quinn D.I., Dorff T.B., Xiong S., Engel J. (2017). A Phase II Trial of AEZS-108 in Castration- and Taxane-Resistant Prostate Cancer. Clin. Genitourin. Cancer.

[191] Kumthekar P., Tang S.C., Brenner A.J., Kesari S., Piccioni D.E., Anders C., Carrillo J., Chalasani P., Kabos P., Puhalla S. (2020). ANG1005, a Brain-Penetrating Peptide-Drug Conjugate, Shows Activity in Patients with Breast Cancer with Leptomeningeal Carcinomatosis and Recurrent Brain Metastases. Clin. Cancer Res..

[192] Demeule M., Charfi C., Currie J.C., Larocque A., Zgheib A., Kozelko S., Béliveau R., Marsolais C., Annabi B. (2021). TH1902, a new docetaxel-peptide conjugate for the treatment of sortilin-positive triple-negative breast cancer. Cancer Sci..

[193] Demeule M., Currie J.C., Charfi C., Zgheib A., Cousineau I., Lullier V., Béliveau R., Marsolais C., Annabi B. (2024). Sudocetaxel Zendusortide (TH1902) triggers the cGAS/STING pathway and potentiates anti-PD-L1 immune-mediated tumor cell killing. Front. Immunol..

[194] Gowland C., Berry P., Errington J., Jeffrey P., Bennett G., Godfrey L., Pittman M., Niewiarowski A., Symeonides S.N., Veal G.J. (2021). Development of a LC-MS/MS method for the quantification of toxic payload DM1 cleaved from BT1718 in a Phase I study. Bioanalysis.

[195] Bashir B., Wang J.S., Falchook G., Fontana E., Arkenau H.T., Carter L., Galot R., Basu B., Greystoke A., Subbiah V. (2024). Results from First-in-Human Phase I Dose-Escalation Study of a Novel Bicycle Toxin Conjugate Targeting EphA2 (BT5528) in Patients with Advanced Solid Tumors. J. Clin. Oncol..

[196] Rigby M., Bennett G., Chen L., Mudd G.E., Harrison H., Beswick P.J., Van Rietschoten K., Watcham S.M., Scott H.S., Brown A.N. (2022). BT8009; A Nectin-4 Targeting Bicycle Toxin Conjugate for Treatment of Solid Tumors. Mol. Cancer Ther..

[197] Su P.L., Chakravarthy K., Furuya N., Brownstein J., Yu J., Long M., Carbone D., Li Z., He K. (2024). DLL3-guided therapies in small-cell lung cancer: From antibody-drug conjugate to precision immunotherapy and radioimmunotherapy. Mol. Cancer.

[198] Wu X., Xu L., Li X., Zhou Y., Han X., Zhang W., Wang W., Guo W., Liu W., Xu Q. (2023). A HER2-targeting antibody-MMAE conjugate RC48 sensitizes immunotherapy in HER2-positive colon cancer by triggering the cGAS-STING pathway. Cell Death Dis..

[199] Yu X., Long Y., Chen B., Tong Y., Shan M., Jia X., Hu C., Liu M., Zhou J., Tang F. (2022). PD-L1/TLR7 dual-targeting nanobody-drug conjugate mediates potent tumor regression via elevating tumor immunogenicity in a host-expressed PD-L1 bias-dependent way. J. Immunother. Cancer.

[200] Levin A., Hakala T.A., Schnaider L., Bernardes G.J.L., Gazit E., Knowles T.P.J. (2020). Biomimetic peptide self-assembly for functional materials. Nat. Rev. Chem..

[201] Yang J., An H.W., Wang H. (2021). Self-Assembled Peptide Drug Delivery Systems. ACS Appl. Bio Mater..

[202] Fleming S., Ulijn R.V. (2014). Design of nanostructures based on aromatic peptide amphiphiles. Chem. Soc. Rev..

[203] Yan X., Zhu P., Li J. (2010). Self-assembly and application of diphenylalanine-based nanostructures. Chem. Soc. Rev..

[204] Sinthuvanich C., Nagy-Smith K.J., Walsh S.T.R., Schneider J.P. (2017). Triggered Formation of Anionic Hydrogels from Self-Assembling Acidic Peptide Amphiphiles. Macromolecules.

[205] Wan W.J., Qu C.X., Zhou Y.J., Zhang L., Chen M.T., Liu Y., You B.G., Li F., Wang D.D., Zhang X.N. (2019). Doxorubicin and siRNA-PD-L1 co-delivery with T7 modified ROS-sensitive nanoparticles fortumor chemoimmunotherapy. Int. J. Pharm..

[206] Ji Y., Xiao Y., Xu L., He J., Qian C., Li W., Wu L., Chen R., Wang J., Hu R. (2018). Drug-Bearing Supramolecular MMP Inhibitor Nanofibers for Inhibition of Metastasis and Growth of Liver Cancer. Adv. Sci..

[207] Yang P., Song H., Qin Y., Huang P., Zhang C., Kong D., Wang W. (2018). Engineering Dendritic-Cell-Based Vaccines and PD-1 Blockade in Self-Assembled Peptide Nanofibrous Hydrogel to Amplify Antitumor T-Cell Immunity. Nano Lett..

[208] Liu D., Wang J., You W., Ma F., Sun Q., She J., He W., Yang G. (2023). A d-peptide-based oral nanotherapeutic modulates the PD-1/PD-L1 interaction for tumor immunotherapy. Front. Immunol..

[209] Song W., Kuang J., Li C.X., Zhang M., Zheng D., Zeng X., Liu C., Zhang X.Z. (2018). Enhanced Immunotherapy Based on Photodynamic Therapy for Both Primary and Lung Metastasis Tumor Eradication. ACS Nano.

[210] Park J., Shin Y., Kim J.M., Kweon S., Song A.Y., Baek Y., Kim J., Cho D., Kim H.S., Doh J. (2021). Multifunctional Microparticles with Stimulation and Sensing Capabilities for Facile NK Cell Activity Assay. ACS Sens..

[211] Moon Y., Shim M.K., Choi J., Yang S., Kim J., Yun W.S., Cho H., Park J.Y., Kim Y., Seong J.K. (2022). Anti-PD-L1 peptide-conjugated prodrug nanoparticles for targeted cancer immunotherapy combining PD-L1 blockade with immunogenic cell death. Theranostics.

[212] Hingorani D.V., Allevato M.M., Camargo M.F., Lesperance J., Quraishi M.A., Aguilera J., Franiak-Pietryga I., Scanderbeg D.J., Wang Z., Molinolo A.A. (2022). Monomethyl auristatin antibody and peptide drug conjugates for trimodal cancer chemo-radio-immunotherapy. Nat. Commun..

[213] Li M., Wang Z., Liu X., Song N., Song Y., Shi X., Liu J., Liu J., Yu Z. (2021). Adaptable peptide-based therapeutics modulating tumor microenvironment for combinatorial radio-immunotherapy. J. Control. Release.

[214] Xiao Q., Huang J., Wang X., Chen Z., Zhang W., Liu F., Li J., Yang Z., Zhan J., Cai Y. (2024). Supramolecular Peptide Amphiphile Nanospheres Reprogram Tumor-associated Macrophage to Reshape the Immune Microenvironment for Enhanced Breast Cancer Immunotherapy. Small.

[215] Lv M.Y., Xiao W.Y., Zhang Y.P., Jin L.L., Li Z.H., Lei Z., Cheng D.B., Jin S.D. (2022). In situ self-assembled peptide enables effective cancer immunotherapy by blockage of CD47. Colloids Surf. B Biointerfaces.

[216] Wu Y., Wen H., Bernstein Z.J., Hainline K.M., Blakney T.S., Congdon K.L., Snyder D.J., Sampson J.H., Sanchez-Perez L., Collier J.H. (2022). Multiepitope supramolecular peptide nanofibers eliciting coordinated humoral and cellular antitumor immune responses. Sci. Adv..

[217] Zhang L., Tian Y., Li M., Wang M., Wu S., Jiang Z., Wang Q., Wang W. (2022). Peptide nano ’bead-grafting’ for SDT-facilitated immune checkpoints blocking. Chem. Sci..

[218] Zhang W., Li D., Xu X., Chen Y., Shi X., Pan Y., Yao S., Piao Y., Zhou Z., Slater N.K.H. (2023). A Bispecific Peptide-Polymer Conjugate Bridging Target-Effector Cells to Enhance Immunotherapy. Adv. Healthc. Mater..

[219] Leach D.G., Dharmaraj N., Lopez-Silva T.L., Venzor J.R., Pogostin B.H., Sikora A.G., Hartgerink J.D., Young S. (2021). Biomaterial-Facilitated Immunotherapy for Established Oral Cancers. ACS Biomater. Sci. Eng..

[220] Wang T., Wang D., Yu H., Feng B., Zhou F., Zhang H., Zhou L., Jiao S., Li Y. (2018). A cancer vaccine-mediated postoperative immunotherapy for recurrent and metastatic tumors. Nat. Commun..

[221] Wu X., Wu Y., Ye H., Yu S., He C., Chen X. (2017). Interleukin-15 and cisplatin co-encapsulated thermosensitive polypeptide hydrogels for combined immuno-chemotherapy. J. Control. Release.

[222] Leach D.G., Dharmaraj N., Piotrowski S.L., Lopez-Silva T.L., Lei Y.L., Sikora A.G., Young S., Hartgerink J.D. (2018). STINGel: Controlled release of a cyclic dinucleotide for enhanced cancer immunotherapy. Biomaterials.

[223] Platten M., Wick W., Van den Eynde B.J. (2012). Tryptophan catabolism in cancer: Beyond IDO and tryptophan depletion. Cancer Res..

[224] Hasannejad-Asl B., Pooresmaeil F., Takamoli S., Dabiri M., Bolhassani A. (2022). Cell penetrating peptide: A potent delivery system in vaccine development. Front. Pharmacol..

[225] Cavallaro P.A., De Santo M., Belsito E.L., Longobucco C., Curcio M., Morelli C., Pasqua L., Leggio A. (2023). Peptides Targeting HER2-Positive Breast Cancer Cells and Applications in Tumor Imaging and Delivery of Chemotherapeutics. Nanomaterials.

[226] Järvinen T.A.H., Pemmari T. (2020). Systemically Administered, Target-Specific, Multi-Functional Therapeutic Recombinant Proteins in Regenerative Medicine. Nanomaterials.

[227] Xing R., Li S., Zhang N., Shen G., Möhwald H., Yan X. (2017). Self-Assembled Injectable Peptide Hydrogels Capable of Triggering Antitumor Immune Response. Biomacromolecules.

[228] Hong E., Dobrovolskaia M.A. (2019). Addressing barriers to effective cancer immunotherapy with nanotechnology: Achievements, challenges, and roadmap to the next generation of nanoimmunotherapeutics. Adv. Drug Deliv. Rev..

[229] Kimura T., Egawa S., Uemura H. (2017). Personalized peptide vaccines and their relation to other therapies in urological cancer. Nat. Rev. Urol..

[230] Scott J.K., Smith G.P. (1990). Searching for peptide ligands with an epitope library. Science.

[231] Lam K.S., Lebl M., Krchnák V. (1997). The “One-Bead-One-Compound” Combinatorial Library Method. Chem. Rev..

[232] Josephson K., Ricardo A., Szostak J.W. (2014). mRNA display: From basic principles to macrocycle drug discovery. Drug Discov. Today.

[233] Lennard K.R., Tavassoli A. (2014). Peptides come round: Using SICLOPPS libraries for early stage drug discovery. Chemistry.

